# Supplementary material for: Drug repurposing for aging research using model organisms
Source: Aging Cell. 2017 Jun 16;16(5):1006–15. doi: 10.1111/acel.12626 (PMC5595691; doi:10.1111/acel.12626)
Supplement: Supplementary file 7 — Data S1 Zip‐Archive of all report cards. [file ACEL-16-1006-s007.zip › RC_ADP.pdf]

ADP

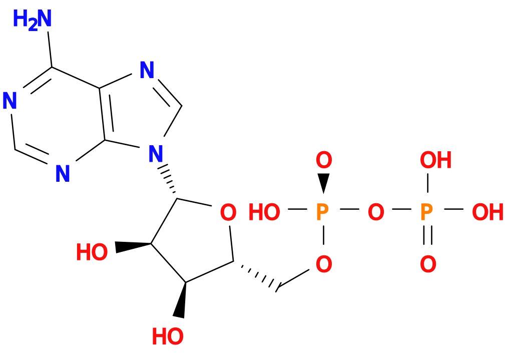

Database identifiers

ChEMBLCompound	CHEMBL14830
CHEBI	16761
eMolecules	474954

Ranking

	Rank	Score
Drosophila	346/697	0.434
C. elegans	572/591	0.0

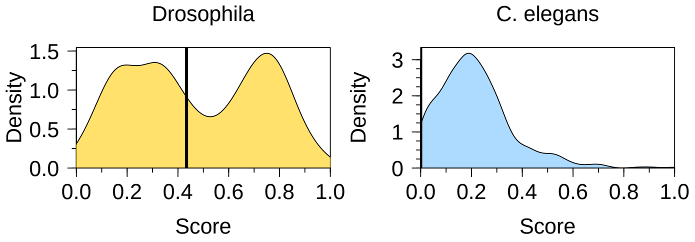

	Ageing implication	Domain conservation	Binding site conservation	Binding affinity	Bioavailability	Lipinski	Promiscuity	Purchasability	Drug approval	**Total**
Drosophila	1.0	0.977	1.0	0.721	(0.9)	-0.1	-0.2	0.1	0.0	0.434
C. elegans	1.0	0.982	1.0	0.721	0.206	-0.1	-0.2	0.1	0.0	0.0

Names

- **ADP**
- 5'-adenylphosphoric acid
- Adenosine-5'-diphosphate
- H3adp

Roles

human metabolite, fundamental metabolite

Status

Approved drug (according to ChEMBL)	No
Number of Rule of 5 violations	2
Binding affinity to original target in log units (RF-Score prediction)	5.95
Burns *C. elegans* bioavailability prediction	-6.93

Compound Target Characteristics

Serine/threonine-protein kinase Chk2

Best gene implication in ageing for this target family came from gene O96017 annotated in UniProt release 2014_02. Annotation GO subterm of 7568 (aging) was Non-traceable Author Statement

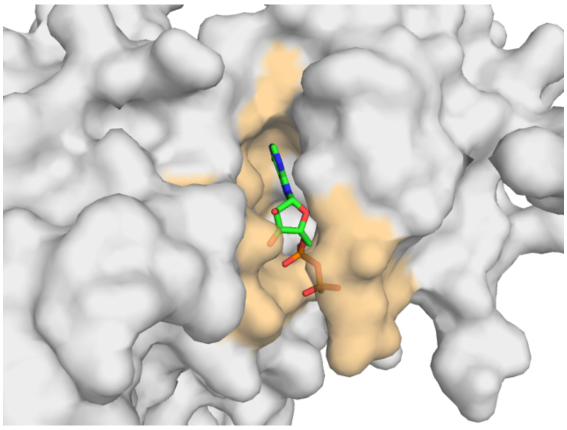

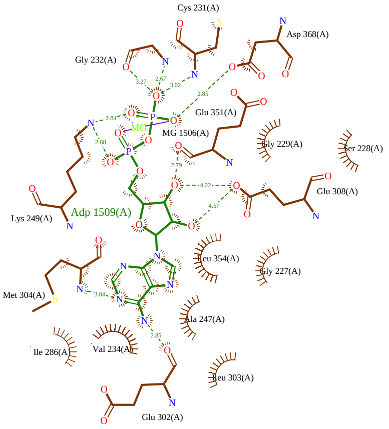

protein	amino acids contacts (binding site)
PDB:2cn5:chainA:O96017	G S G A C G V A K I E L M E E N L D
sp:O96017:CHK2_HUMAN	G S G A C G V A K I E L M E E N L D
tr:Q9R019:Q9R019_RAT	G S G A C G V A K I E L M E E N L D
sp:Q9Z265:CHK2_MOUSE	G S G A C G V A K I E L M E E N L D
tr:Q543W6:Q543W6_MOUSE	G S G A C G V A K I E L M E E N L D
tr:A4V0X0:A4V0X0_DROME	G S G A Y G V A K V E F M D D N L D
sp:O61267:LOK_DROME	G S G A Y G V A K V E F M D D N L D
tr:A4F324:A4F324_CAEEL	G K G G F G V A K V E Y V E E N L D
sp:Q9U1Y5:CHK2_CAEEL	G K G G F G V A K V E Y V E E N L D
sp:P22216:RAD53_YEAST	G Q G A F A V A K V E F V D D N L D

	whole protein		domain-based		contact-based	
protein	ident	simil	ident	simil	ident	simil
PDB:2cn5:chainA:O96017	0.99	1.0	1.0	1.0	1.0	1.0
sp:O96017:CHK2_HUMAN	1.0	1.0	1.0	1.0	1.0	1.0
tr:Q9R019:Q9R019_RAT	0.82	0.92	0.92	0.98	1.0	1.0
sp:Q9Z265:CHK2_MOUSE	0.82	0.92	0.92	0.98	1.0	1.0
tr:Q543W6:Q543W6_MOUSE	0.82	0.92	0.92	0.98	1.0	1.0
tr:A4V0X0:A4V0X0_DROME	0.31	0.65	0.43	0.81	0.72	0.83
sp:O61267:LOK_DROME	0.3	0.63	0.43	0.81	0.72	0.83
tr:A4F324:A4F324_CAEEL	0.27	0.62	0.36	0.75	0.67	0.76
sp:Q9U1Y5:CHK2_CAEEL	0.27	0.62	0.36	0.75	0.67	0.76
sp:P22216:RAD53_YEAST	0.16	0.4	0.38	0.73	0.56	0.74

lok (FBgn0019686) associated phenotypes

cell death defective, chemical resistant, conditional, decreased cell death, increased cell death, long lived, maternal effect, radiation sensitive

(Information from FlyBase)

lok (UniProt:O61267) annotation

Function: May have a role in germline establishment. (PubMed:9507063).

Subcellular location: Nucleus speckle(PubMed:9507063). Note=Speckled subnuclear compartment.

Tissue specificity: In stage 3 embryos, both isoforms are expressed in both somatic and pole cell nuclei. Expression in pole cell nuclei is sustained until stage 9 and weakly expressed after pole cell invagination into the abdominal cavity. (PubMed:9507063).

Developmental stage: Expressed both maternally and zygotically in adult females. Levels of the long isoform remain fairly constant from ovaries to embryos, the levels of short isoform decrease dramatically. (PubMed:9507063).

(Information from UniProt)

chk-2 (UniProt:Q9U1Y5) annotation

Function: Serine/threonine-protein kinase which is required for checkpoint-mediated cell cycle arrest, activation of DNA repair and apoptosis in response to the presence of DNA double-strand breaks. May also negatively regulate cell cycle progression during unperturbed cell cycles. Phosphorylates and inhibits cdc25 phosphatase, preventing entry into mitosis. Required for nuclear reorganization and homologous chromosome pairing during meiotic prophase. (PubMed:11086161, PubMed:11158318).

Cofactor: Mg(2+)

Subcellular location: Nucleus (PubMed:11086161, PubMed:11158318).

Tissue specificity: Highly expressed in germline tissue. (PubMed:11086161).

Developmental stage: Low expression in embryos and L2 larvae, high expression in hermaphrodite adults. (PubMed:11086161).

(Information from UniProt)

Tyrosine-protein kinase ABL1

Best gene implication in ageing for this target family came from gene P00519 via mapping the annotation from Ensembl ENSG00000097007 via mapping the annotation from EntrezGene 25 via mapping the annotation from GenAgeHuman 0078 annotated in GenAge release 17.

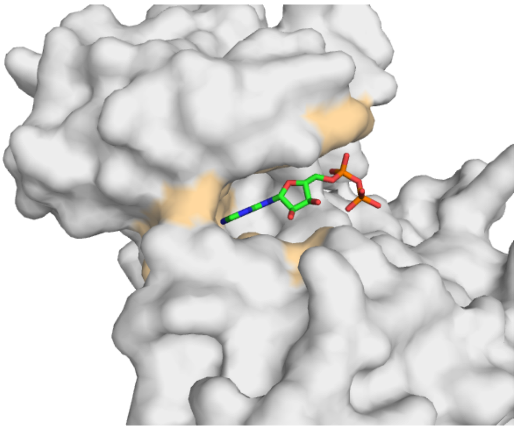

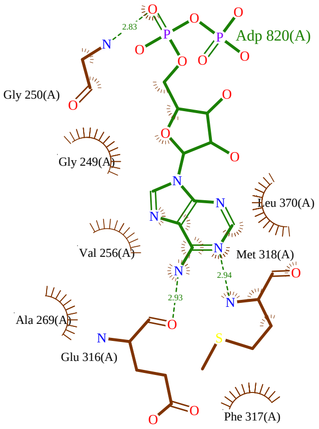

protein	amino acids contacts (binding site)
PDB:2g2i:chainA:P00519	G G V A T E F M N L
sp:P00519:ABL1_HUMAN	G G V A T E F M N L
tr:Q59FK4:Q59FK4_HUMAN	G G V A T E F M N L
tr:F1M0A6:F1M0A6_RAT	G G V A T E F M N L
tr:D3ZGM3:D3ZGM3_RAT	G G V A T E F M N L
tr:E9PT20:E9PT20_RAT	G G V A T E F M N L
tr:Q3V2W1:Q3V2W1_MOUSE	G G V A T E F M N L
sp:P00520:ABL1_MOUSE	G G V A T E F M N L
tr:Q3SYK5:Q3SYK5_MOUSE	G G V A T E F M N L
sp:P00522:ABL_DROME	G G V A T E F M N L
tr:A8JNU2:A8JNU2_DROME	G G V A T E F M N L
sp:P03949:ABL1_CAEEL	G G V A T E F M N L

	whole protein		domain-based		contact-based	
protein	ident	simil	ident	simil	ident	simil
PDB:2g2i:chainA:P00519	1.0	1.0	1.0	1.0	1.0	1.0
sp:P00519:ABL1_HUMAN	1.0	1.0	1.0	1.0	1.0	1.0
tr:Q59FK4:Q59FK4_HUMAN	0.95	0.96	1.0	1.0	1.0	1.0
tr:F1M0A6:F1M0A6_RAT	0.84	0.93	1.0	1.0	1.0	1.0
tr:D3ZGM3:D3ZGM3_RAT	0.87	0.95	1.0	1.0	1.0	1.0
tr:E9PT20:E9PT20_RAT	0.84	0.93	1.0	1.0	1.0	1.0
tr:Q3V2W1:Q3V2W1_MOUSE	0.3	0.32	1.0	1.0	1.0	1.0
sp:P00520:ABL1_MOUSE	0.87	0.95	1.0	1.0	1.0	1.0
tr:Q3SYK5:Q3SYK5_MOUSE	0.84	0.93	1.0	1.0	1.0	1.0
sp:P00522:ABL_DROME	0.26	0.49	0.78	0.93	1.0	1.0
tr:A8JNU2:A8JNU2_DROME	0.25	0.48	0.78	0.93	1.0	1.0
sp:P03949:ABL1_CAEEL	0.31	0.62	0.67	0.9	1.0	1.0

Abl (FBgn0000017) associated phenotypes

cell migration defective, cell shape defective, germline clone, heat sensitive, lethal - all die before end of pupal stage, maternal effect, mitotic cell cycle defective, neuroanatomy defective, neurophysiology defective, partially lethal - majority die, partially lethal - majority live, reduced, rescuable maternal effect, size defective, somatic clone, some die during pupal stage

(Information from FlyBase)

Abl (UniProt:P00522) annotation

Function: Arm and Abl proteins function cooperatively at adherens junctions in both the CNS and epidermis; critical for embryonic epithelial morphogenesis regulating cell shape changes and cell migration. Plays a critical role in transducing embryonic midline repulsive cues; may regulate cytoskeletal dynamics underlying a growth cone's response to midline cues. The ability of pCC/MP2 axons to correctly interpret midline repulsive cues and stay on the ipsilateral side is dependent on the strength of both Slit/robo and Abl-dependent signaling pathways. (PubMed:11756472, PubMed:12973825, PubMed:9635189).

Subcellular location: Cytoplasm.

Tissue specificity: Arm and ena colocalize with Abl at adherens junctions throughout development. (PubMed:11756472, PubMed:9635189).

Developmental stage: Expressed both maternally and zygotically. (PubMed:2832740).

Disruption phenotype: Both loss- and gain-of-function mutants exhibit neurons within the pCC/MP2 pathway that incorrectly project across the midline. Loss of Abl disrupts cell migration and cell shape changes during dorsal closure. (PubMed:11756472, PubMed:9635189).

(Information from UniProt)

abl-1 (WBGene00000018) associated phenotypes

apoptosis variant, gamma ray induced apoptosis increased, lipid synthesis increased, protein subcellular localization variant

(Information from WormBase)

ATP synthase subunit beta, mitochondrial

Best gene implication in ageing for this target family came from gene Q8T4C4 annotated in UniProt release 2014_02. Annotation GO 8340 (determination of adult lifespan) was Inferred from Mutant Phenotype

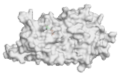

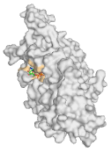

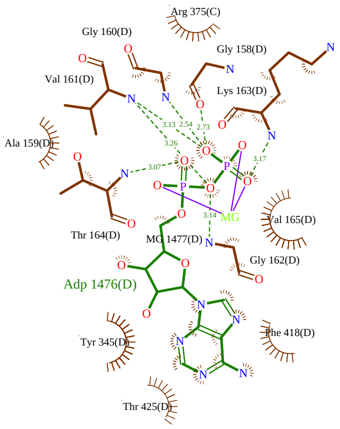

protein	amino acids contacts (binding site)
PDB:2wpd:chainD:P00830	G A G V G K T V Y F A F T
PDB:3zia:chainD:P00830	G A G V G K T V Y F A F T
PDB:4b2q:chainD:P00830	G A G V G K T V Y F A F T
tr:Q8T4C4:Q8T4C4_DROME	G A G V G K T V Y F A F T
sp:P00830:ATPB_YEAST	G A G V G K T V Y F A F T

	whole protein		domain-based		contact-based	
protein	ident	simil	ident	simil	ident	simil
PDB:2wpd:chainD:P00830	1.0	1.0	1.0	1.0	1.0	1.0
PDB:3zia:chainD:P00830	1.0	1.0	1.0	1.0	1.0	1.0
PDB:4b2q:chainD:P00830	1.0	1.0	1.0	1.0	1.0	1.0
tr:Q8T4C4:Q8T4C4_DROME	0.55	0.73	0.75	0.92	1.0	1.0
sp:P00830:ATPB_YEAST	1.0	1.0	1.0	1.0	1.0	1.0

ms(3)72Dt (FBgn0036568) associated phenotypes

RU486 conditional, calorie restriction conditional, female semi-fertile, long lived, short lived

(Information from FlyBase)

ms(3)72Dt (UniProt:Q8T4C4) annotation

Function: Produces ATP from ADP in the presence of a proton gradient across the membrane. (RuleBase:RU003553).

Subunit: F-type ATPases have 2 components, CF(1) - the catalytic core - and CF(0) - the membrane proton channel. CF(1) has five subunits: alpha(3), beta(3), gamma(1), delta(1), epsilon(1). CF(0) has three main subunits: a, b and c. (RuleBase:RU004290).

(Information from UniProt)

ATP-dependent molecular chaperone HSP82

Best gene implication in ageing for this target family came from gene Q18688 annotated in UniProt release 2014_02. Annotation GO 8340 (determination of adult lifespan) was Inferred from Genetic Interaction

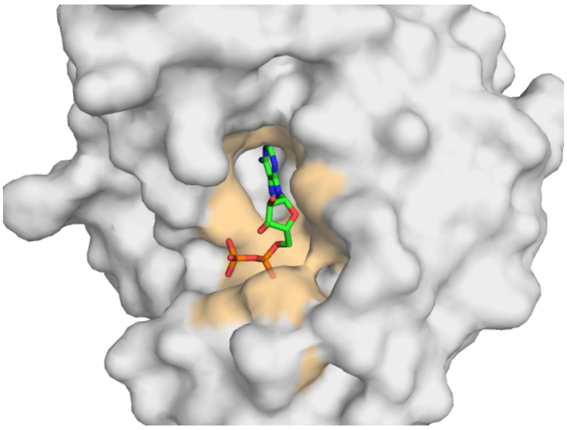 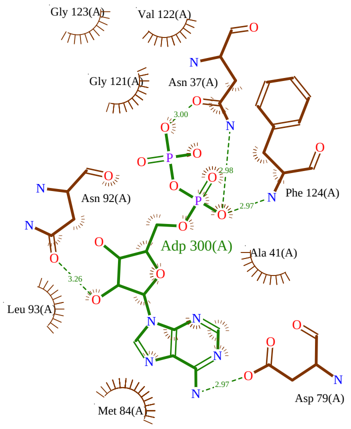

protein	amino acids contacts (binding site)											
PDB:1am1:chainA:P02829	N	A	D	M	N	L	K	G	V	G	F	T
PDB:1amw:chainA:P02829	N	A	D	M	N	L	K	G	V	G	F	T
PDB:2wep:chainA:P02829	N	A	D	M	N	L	K	G	V	G	F	T
tr:K9JA46:K9JA46_HUMAN	N	A	D	M	N	L	K	G	V	G	F	T
sp:P07900:HS90A_HUMAN	N	A	D	M	N	L	K	G	V	G	F	T
tr:Q6B437:Q6B437_RAT	N	A	D	M	N	L	K	G	V	G	F	T
sp:P82995:HS90A_RAT	N	A	D	M	N	L	K	G	V	G	F	T
tr:B7ZC50:B7ZC50_MOUSE	N	A	D	M	N	L	K	G	V	G	F	T
tr:Q8C2A9:Q8C2A9_MOUSE	N	A	D	M	N	L	K	G	V	G	F	T
tr:Q3TKB9:Q3TKB9_MOUSE	N	A	D	M	N	L	K	G	V	G	F	T
tr:Q3TKG0:Q3TKG0_MOUSE	N	A	D	M	N	L	K	G	V	G	F	T
sp:P07901:HS90A_MOUSE	N	A	D	M	N	L	K	G	V	G	F	T
tr:Q80Y52:Q80Y52_MOUSE	N	A	D	M	N	L	K	G	V	G	F	T
sp:P02828:HSP83_DROME	N	A	D	M	N	L	K	G	V	G	F	T
sp:Q18688:HSP90_CAEEL	N	A	D	M	N	L	K	G	V	G	F	T
sp:P02829:HSP82_YEAST	N	A	D	M	N	L	K	G	V	G	F	T

	whole protein		domain-based		contact-based	
protein	ident	simil	ident	simil	ident	simil
PDB:1am1:chainA:P02829	1.0	1.0	1.0	1.0	1.0	1.0
PDB:1amw:chainA:P02829	1.0	1.0	1.0	1.0	1.0	1.0
PDB:2wep:chainA:P02829	0.99	1.0	0.99	1.0	1.0	1.0
tr:K9JA46:K9JA46_HUMAN	0.6	0.85	0.68	0.9	1.0	1.0
sp:P07900:HS90A_HUMAN	0.6	0.85	0.68	0.9	1.0	1.0
tr:Q6B437:Q6B437_RAT	0.16	0.21	0.58	0.75	1.0	1.0
sp:P82995:HS90A_RAT	0.6	0.85	0.68	0.9	1.0	1.0
tr:B7ZC50:B7ZC50_MOUSE	0.18	0.23	0.59	0.77	1.0	1.0
tr:Q8C2A9:Q8C2A9_MOUSE	0.23	0.31	0.68	0.9	1.0	1.0
tr:Q3TKB9:Q3TKB9_MOUSE	0.48	0.65	0.68	0.9	1.0	1.0
tr:Q3TKG0:Q3TKG0_MOUSE	0.48	0.66	0.68	0.9	1.0	1.0
sp:P07901:HS90A_MOUSE	0.6	0.85	0.68	0.9	1.0	1.0
tr:Q80Y52:Q80Y52_MOUSE	0.6	0.85	0.68	0.9	1.0	1.0
sp:P02828:HSP83_DROME	0.6	0.86	0.67	0.9	1.0	1.0
sp:Q18688:HSP90_CAEEL	0.6	0.84	0.66	0.9	1.0	1.0
sp:P02829:HSP82_YEAST	1.0	1.0	1.0	1.0	1.0	1.0

Hsp83 (FBgn0001233) associated phenotypes

cell cycle defective, cell polarity defective, developmental rate defective, female semi-fertile, lethal - all die before end of pupal stage, lethal - all die during third instar larval stage, partially lethal, partially lethal - majority die, short lived

(Information from FlyBase)

Hsp83 (UniProt:P02828) annotation

Function: Molecular chaperone that promotes the maturation, structural maintenance and proper regulation of specific target proteins involved for instance in cell cycle control and signal trans-

duction. Undergoes a functional cycle that is linked to its ATPase activity. This cycle probably induces conformational changes in the client proteins, thereby causing their activation. Interacts dynamically with various co-chaperones that modulate its substrate recognition, ATPase cycle and chaperone function. Together with Hop and piwi, mediates canalization, also known as developmental robustness, likely via epigenetic silencing of existing genetic variants and suppression of transposon-induced new genetic variation. Required for piRNA biogenesis by facilitating loading of piRNAs into PIWI proteins. (PubMed:21186352, PubMed:22902557).
Subunit: Homodimer (By similarity). Forms a complex with Hop and piwi; probably Hop mediates the interaction between piwi and Hsp83. Interacts with shu. (, PubMed:21186352, PubMed:22902557).
Subcellular location: Cytoplasm.
Induction: In contrast to other major heat shock proteins, this one is also expressed at normal growth temperatures. It is also developmentally expressed during oogenesis.
Domain: The TPR repeat-binding motif mediates interaction with TPR repeat-containing proteins.

(Information from UniProt)

daf-21 (WBGene00000915) associated phenotypes
chemotaxis variant, dauer constitutive, dauer recovery variant, larval lethal, lethal, odorant chemosensory response variant, sluggish, sterile

(Information from WormBase)

daf-21 (UniProt:Q18688) annotation
Function: Molecular chaperone that promotes the maturation, structural maintenance and proper regulation of specific target proteins involved for instance in cell cycle control and signal transduction. Undergoes a functional cycle that is linked to its ATPase activity. This cycle probably induces conformational changes in the client proteins, thereby causing their activation. Interacts dynamically with various co-chaperones that modulate its substrate recognition, ATPase cycle and chaperone function. By stabilizing the receptor-type guanylate cyclase daf-11 or another signal transduction component that regulates cGMP levels, plays a role in dauer formation and chemotaxis to non-volatile and volatile attractants detected by AWC sensory neurons (PubMed:10790386, PubMed:7828815). Participates in the control of cell cycle progression at the prophase/metaphase transition in oocyte development by ensuring the activity of wee-1.3 kinase, which negatively regulates cdk-1 through its phosphorylation (PubMed:16466390). Regulates yap-1 nuclear export after heat shock treatment (PubMed:23396260). (PubMed:10790386, PubMed:16466390, PubMed:23396260, PubMed:7828815).
Subunit: Homodimer. (UniProtKB:P08238).
Subcellular location: Cytoplasm, perinuclear region (PubMed:12950278). Note=Perinuclear region of somatic cells.
Tissue specificity: In the embryo comma stage, expression is strongly detected in cells of the head region and less so in other areas. In early larvae, expressed in postembryonic germ cells derived from Z2 and Z3 cells and the head region, in both hermaphrodites and males. Under heat stress conditions, larval expression is not only detected in germ cells, but also all over the body. In adult hermaphrodites, expression is localized uniquely in the germ cells. (PubMed:12950278).
Developmental stage: Highly expressed throughout development. (PubMed:10790386).
Domain: The TPR repeat-binding motif mediates interaction with TPR repeat-containing proteins.

(Information from UniProt)

Heat shock protein HSP 90-alpha

Best gene implication in ageing for this target family came from gene Q18688 annotated in UniProt release 2014_02. Annotation GO 8340 (determination of adult lifespan) was Inferred from Genetic

Interaction

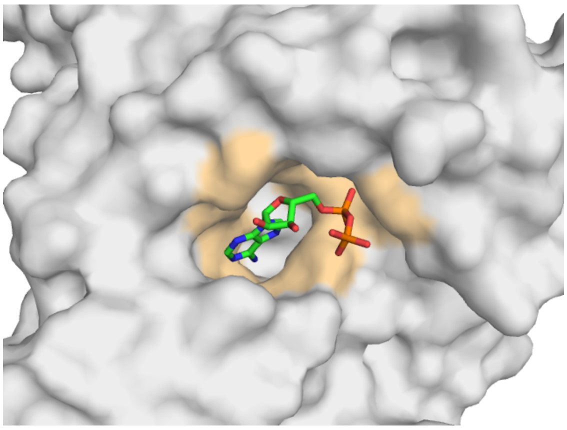

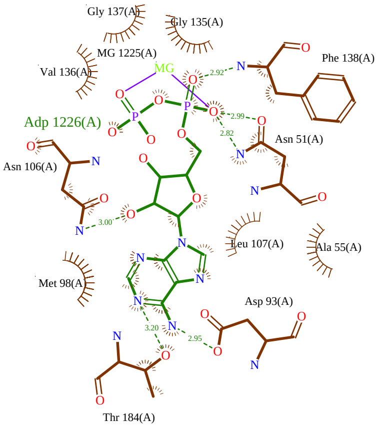

protein	amino acids contacts (binding site)
PDB:1byq:chainA:P07900	N A D M N L G V G F T
PDB:2xk2:chainA:P07900	N A D M N L G V G F T
tr:K9JA46:K9JA46_HUMAN	N A D M N L G V G F T
sp:P07900:HS90A_HUMAN	N A D M N L G V G F T
tr:Q6B437:Q6B437_RAT	N A D M N L G V G F T
sp:P82995:HS90A_RAT	N A D M N L G V G F T
tr:B7ZC50:B7ZC50_MOUSE	N A D M N L G V G F T
tr:Q8C2A9:Q8C2A9_MOUSE	N A D M N L G V G F T
tr:Q3TKB9:Q3TKB9_MOUSE	N A D M N L G V G F T
tr:Q3TKG0:Q3TKG0_MOUSE	N A D M N L G V G F T
sp:P07901:HS90A_MOUSE	N A D M N L G V G F T
tr:Q80Y52:Q80Y52_MOUSE	N A D M N L G V G F T
sp:P02828:HSP83_DROME	N A D M N L G V G F T
sp:Q18688:HSP90_CAEEL	N A D M N L G V G F T
sp:P02829:HSP82_YEAST	N A D M N L G V G F T

	whole protein		domain-based		contact-based	
protein	ident	simil	ident	simil	ident	simil
PDB:1byq:chainA:P07900	1.0	1.0	0.99	1.0	1.0	1.0
PDB:2xk2:chainA:P07900	1.0	1.0	1.0	1.0	1.0	1.0
tr:K9JA46:K9JA46_HUMAN	1.0	1.0	1.0	1.0	1.0	1.0
sp:P07900:HS90A_HUMAN	1.0	1.0	1.0	1.0	1.0	1.0
tr:Q6B437:Q6B437_RAT	0.22	0.22	0.85	0.85	1.0	1.0
sp:P82995:HS90A_RAT	0.99	1.0	1.0	1.0	1.0	1.0
tr:B7ZC50:B7ZC50_MOUSE	0.27	0.27	0.86	0.86	1.0	1.0
tr:Q8C2A9:Q8C2A9_MOUSE	0.37	0.37	0.99	1.0	1.0	1.0
tr:Q3TKB9:Q3TKB9_MOUSE	0.75	0.76	0.99	1.0	1.0	1.0
tr:Q3TKG0:Q3TKG0_MOUSE	0.75	0.76	0.99	1.0	1.0	1.0
sp:P07901:HS90A_MOUSE	0.99	1.0	0.99	1.0	1.0	1.0
tr:Q80Y52:Q80Y52_MOUSE	0.99	1.0	0.99	1.0	1.0	1.0
sp:P02828:HSP83_DROME	0.77	0.92	0.85	0.96	1.0	1.0
sp:Q18688:HSP90_CAEEL	0.74	0.89	0.76	0.94	1.0	1.0
sp:P02829:HSP82_YEAST	0.6	0.84	0.68	0.9	1.0	1.0

Hsp83 (FBgn0001233) associated phenotypes

cell cycle defective, cell polarity defective, developmental rate defective, female semi-fertile, lethal - all die before end of pupal stage, lethal - all die during third instar larval stage, partially lethal, partially lethal - majority die, short lived

(Information from FlyBase)

Hsp83 (UniProt:P02828) annotation

Function: Molecular chaperone that promotes the maturation, structural maintenance and proper regulation of specific target proteins involved for instance in cell cycle control and signal trans-

duction. Undergoes a functional cycle that is linked to its ATPase activity. This cycle probably induces conformational changes in the client proteins, thereby causing their activation. Interacts dynamically with various co-chaperones that modulate its substrate recognition, ATPase cycle and chaperone function. Together with Hop and piwi, mediates canalization, also known as developmental robustness, likely via epigenetic silencing of existing genetic variants and suppression of transposon-induced new genetic variation. Required for piRNA biogenesis by facilitating loading of piRNAs into PIWI proteins. (PubMed:21186352, PubMed:22902557).
Subunit: Homodimer (By similarity). Forms a complex with Hop and piwi; probably Hop mediates the interaction between piwi and Hsp83. Interacts with shu. (, PubMed:21186352, PubMed:22902557).
Subcellular location: Cytoplasm.
Induction: In contrast to other major heat shock proteins, this one is also expressed at normal growth temperatures. It is also developmentally expressed during oogenesis.
Domain: The TPR repeat-binding motif mediates interaction with TPR repeat-containing proteins.

(Information from UniProt)

daf-21 (WBGene00000915) associated phenotypes
chemotaxis variant, dauer constitutive, dauer recovery variant, larval lethal, lethal, odorant chemosensory response variant, sluggish, sterile

(Information from WormBase)

daf-21 (UniProt:Q18688) annotation
Function: Molecular chaperone that promotes the maturation, structural maintenance and proper regulation of specific target proteins involved for instance in cell cycle control and signal transduction. Undergoes a functional cycle that is linked to its ATPase activity. This cycle probably induces conformational changes in the client proteins, thereby causing their activation. Interacts dynamically with various co-chaperones that modulate its substrate recognition, ATPase cycle and chaperone function. By stabilizing the receptor-type guanylate cyclase daf-11 or another signal transduction component that regulates cGMP levels, plays a role in dauer formation and chemotaxis to non-volatile and volatile attractants detected by AWC sensory neurons (PubMed:10790386, PubMed:7828815). Participates in the control of cell cycle progression at the prophase/metaphase transition in oocyte development by ensuring the activity of wee-1.3 kinase, which negatively regulates cdk-1 through its phosphorylation (PubMed:16466390). Regulates yap-1 nuclear export after heat shock treatment (PubMed:23396260). (PubMed:10790386, PubMed:16466390, PubMed:23396260, PubMed:7828815).
Subunit: Homodimer. (UniProtKB:P08238).
Subcellular location: Cytoplasm, perinuclear region (PubMed:12950278). Note=Perinuclear region of somatic cells.
Tissue specificity: In the embryo comma stage, expression is strongly detected in cells of the head region and less so in other areas. In early larvae, expressed in postembryonic germ cells derived from Z2 and Z3 cells and the head region, in both hermaphrodites and males. Under heat stress conditions, larval expression is not only detected in germ cells, but also all over the body. In adult hermaphrodites, expression is localized uniquely in the germ cells. (PubMed:12950278).
Developmental stage: Highly expressed throughout development. (PubMed:10790386).
Domain: The TPR repeat-binding motif mediates interaction with TPR repeat-containing proteins.

(Information from UniProt)

Heat shock 70 kDa protein 1A

Best gene implication in ageing for this target family came from gene O97125 annotated in UniProt release 2014_02. Annotation GO 8340 (determination of adult lifespan) was Inferred from Mutant

Phenotype

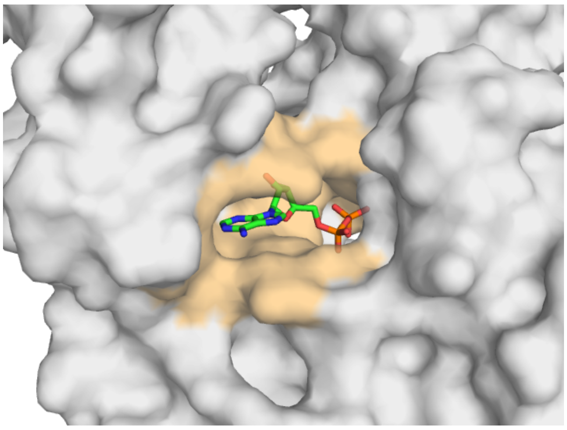

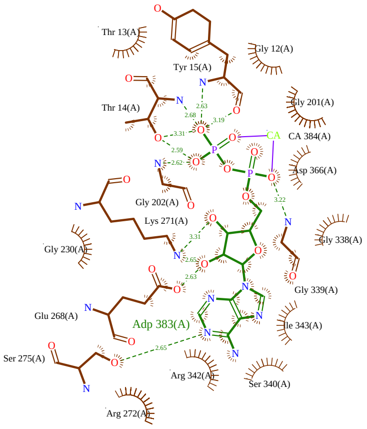

protein	amino acids contacts (binding site)
PDB:1hjo:chainA:P08107	G T T Y G G G G E K R S G G S R I D
PDB:1s3x:chainA:P08107	G T T Y G G G G E K R S G G S R I D
PDB:3atu:chainA:P08107	G T T Y G G G G E K R S G G S R I D
PDB:3atv:chainA:P08107	G T T Y G G G G E K R S G G S R I D
PDB:3ay9:chainA:P08107	G T T Y G G G G E K R S G G S R I D
PDB:3jxu:chainA:P08107	G T T Y G G G G E K R S G G S R I D
tr:E7EP94:E7EP94_HUMAN	G T T Y G G G G E K R S G G S R I D
tr:E9PKE3:E9PKE3_HUMAN	G T T Y G G G G E K R S G G S R I D
sp:P08107:HSP71_HUMAN	G T T Y G G G G E K R S G G S R I D
tr:A8K5I0:A8K5I0_HUMAN	G T T Y G G G G E K R S G G S R I D
sp:P11142:HSP7C_HUMAN	G T T Y G G G G E K R S G G S R I D
sp:Q07439:HSP71_RAT	G T T Y G G G G E K R S G G S R I D
sp:Q61696:HS71A_MOUSE	G T T Y G G G G E K R S G G S R I D
sp:P17879:HS71B_MOUSE	G T T Y G G G G E K R S G G S R I D
tr:Q3TU85:Q3TU85_MOUSE	G T T Y G G G G E K R S G G S R I D
sp:O97125:HSP68_DROME	G T T Y G G G G E K R S G G S R I D
sp:P29843:HSP7A_DROME	G T T Y G G G G E K R S G G S R I D
tr:F3YDI6:F3YDI6_DROME	G T T Y G G G G E K R S G G S R I D
sp:P82910:HSP70_DROME	G T T Y G G G G E K R S G G S R I D
sp:P02825:HSP71_DROME	G T T Y G G G G E K R S G G S R I D
tr:C7LA76:C7LA76_DROME	G T T Y G G G G E K R S G G S R I D
sp:P29844:HSP7C_DROME	G T T Y G G G G E K R S G G S R I D
tr:F3YDH0:F3YDH0_DROME	G T T Y G G G G E K R S G G S R I D
tr:C7LA75:C7LA75_DROME	G T T Y G G G G E K R S G G S R I D
sp:P11147:HSP7D_DROME	G T T Y G G G G E K R S G G S R I D
tr:F3YDH5:F3YDH5_DROME	G T T Y G G G G E K R S G G S R I D
tr:Q8IH88:Q8IH88_DROME	G T T Y G G G G E K R S G G S R I D
sp:P09446:HSP7A_CAEEL	G T T Y G G G G E K R S G G S R I D
sp:P10592:HSP72_YEAST	G T T Y G G G G E K R S G G S R I D

protein	whole protein ident	simil	domain-based ident	simil	contact-based ident	simil
PDB:1hjo:chainA:P08107	1.0	1.0	1.0	1.0	1.0	1.0
PDB:1s3x:chainA:P08107	1.0	1.0	1.0	1.0	1.0	1.0
PDB:3atu:chainA:P08107	1.0	1.0	1.0	1.0	1.0	1.0
PDB:3atv:chainA:P08107	1.0	1.0	1.0	1.0	1.0	1.0
PDB:3ay9:chainA:P08107	1.0	1.0	1.0	1.0	1.0	1.0
PDB:3jxu:chainA:P08107	1.0	1.0	1.0	1.0	1.0	1.0
tr:E7EP94:E7EP94_HUMAN	0.86	0.86	0.87	0.87	1.0	1.0
tr:E9PKE3:E9PKE3_HUMAN	0.82	0.92	0.85	0.92	1.0	1.0
sp:P08107:HSP71_HUMAN	1.0	1.0	1.0	1.0	1.0	1.0
tr:A8K5I0:A8K5I0_HUMAN	1.0	1.0	1.0	1.0	1.0	1.0
sp:P11142:HSP7C_HUMAN	0.85	0.95	0.9	0.97	1.0	1.0
sp:Q07439:HSP71_RAT	0.97	0.99	0.98	1.0	1.0	1.0
sp:Q61696:HS71A_MOUSE	0.95	0.99	0.98	0.99	1.0	1.0
sp:P17879:HS71B_MOUSE	0.95	0.99	0.98	0.99	1.0	1.0
tr:Q3TU85:Q3TU85_MOUSE	0.95	0.99	0.98	0.99	1.0	1.0
sp:O97125:HSP68_DROME	0.72	0.91	0.82	0.95	1.0	1.0
sp:P29843:HSP7A_DROME	0.79	0.94	0.85	0.97	1.0	1.0
tr:F3YDI6:F3YDI6_DROME	0.75	0.91	0.82	0.95	1.0	1.0
sp:P82910:HSP70_DROME	0.75	0.91	0.82	0.95	1.0	1.0
sp:P02825:HSP71_DROME	0.75	0.91	0.82	0.95	1.0	1.0
tr:C7LA76:C7LA76_DROME	0.75	0.91	0.82	0.95	1.0	1.0
sp:P29844:HSP7C_DROME	0.57	0.82	0.68	0.9	1.0	1.0
tr:F3YDH0:F3YDH0_DROME	0.57	0.82	0.68	0.9	1.0	1.0
tr:C7LA75:C7LA75_DROME	0.81	0.94	0.87	0.97	1.0	1.0
sp:P11147:HSP7D_DROME	0.81	0.94	0.87	0.97	1.0	1.0
tr:F3YDH5:F3YDH5_DROME	0.76	0.91	0.85	0.97	1.0	1.0
tr:Q8IH88:Q8IH88_DROME	0.74	0.89	0.85	0.97	1.0	1.0
sp:P09446:HSP7A_CAEEL	0.82	0.94	0.85	0.96	1.0	1.0
sp:P10592:HSP72_YEAST	0.73	0.9	0.8	0.95	1.0	1.0

Hsp68 (FBgn0001230) associated phenotypes

chemical resistant, long lived, neuroanatomy defective, partially lethal - majority die, some die during pupal stage, starvation stress response defective

(Information from FlyBase)

Hsc70-1 (FBgn0001216) associated phenotypes

partially lethal - majority die, some die during pupal stage

(Information from FlyBase)

Hsc70-1 (UniProt:P29843) annotation

Developmental stage: Heat shock cognate proteins are expressed constitutively during normal development.

(Information from UniProt)

Hsp70Aa (UniProt:P82910) annotation

Induction: Heat shock induces the synthesis of seven proteins at five otherwise inactive sites in the polytene chromosomes of fruit fly larvae. Two separate sites, producing two and three copies, respectively, code for the 70 kDa protein.
Miscellaneous: There are two copies of the gene coding for this protein at chromosome locus 87A7.

(Information from UniProt)

Hsp70Ab (UniProt:P02825) annotation

Induction: Heat shock induces the synthesis of seven proteins at five otherwise inactive sites in the polytene chromosomes of fruit fly larvae. Two separate sites, producing two and three copies, respectively, code for the 70 kDa protein.
Miscellaneous: There are two copies of the gene coding for this protein at chromosome locus 87A7.

(Information from UniProt)

Hsc70-3 (FBgn0001218) associated phenotypes
dominant, neuroanatomy defective, partially lethal - majority die

(Information from FlyBase)

Hsc70-3 (UniProt:P29844) annotation
Function: Probably plays a role in facilitating the assembly of multimeric protein complexes inside the ER.
Subcellular location: Endoplasmic reticulum lumen.

(Information from UniProt)

Hsc70-4 (UniProt:P11147) annotation
Subcellular location: Cytoplasm, perinuclear region. Nucleus. Note=Localized to a meshwork of cytoplasmic fibers around the nucleus. Translocates to the nucleus after thermal stress.
Developmental stage: Heat shock cognate proteins are expressed constitutively during normal development.

(Information from UniProt)

hsp-1 (WBGene00002005) associated phenotypes
lethal, sterile

(Information from WormBase)

Heat shock cognate 71 kDa protein

Best gene implication in ageing for this target family came from gene O97125 annotated in UniProt release 2014_02. Annotation GO 8340 (determination of adult lifespan) was Inferred from Mutant Phenotype

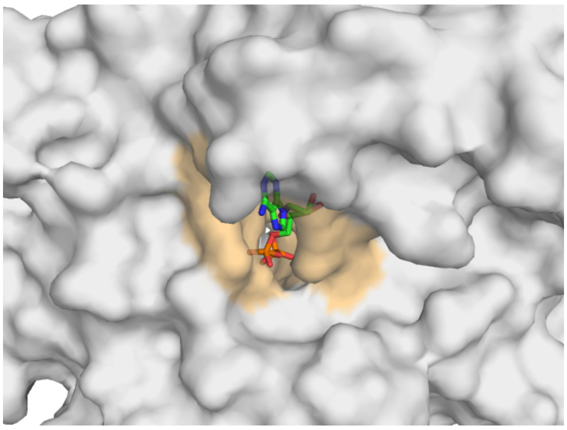

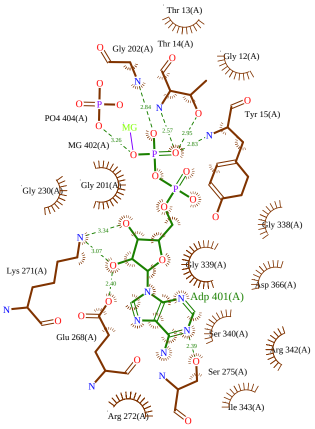

protein	amino acids contacts (binding site)
PDB:4h5t:chainA:P11142	G T T Y G G G E K R S G G S R I D
tr:E7EP94:E7EP94_HUMAN	G T T Y G G G E K R S G G S R I D
tr:E9PKE3:E9PKE3_HUMAN	G T T Y G G G E K R S G G S R I D
sp:P08107:HSP71_HUMAN	G T T Y G G G E K R S G G S R I D
sp:P11142:HSP7C_HUMAN	G T T Y G G G E K R S G G S R I D
tr:A8K5I0:A8K5I0_HUMAN	G T T Y G G G E K R S G G S R I D
tr:M0R8M9:M0R8M9_RAT	G T T Y G G G E K R S G G S R I D
tr:F1LZI1:F1LZI1_RAT	G T T Y G G G E K R S G G S R I D
tr:M0RCB1:M0RCB1_RAT	G T T Y G G G E K R S G G S R I D
tr:Q3TB63:Q3TB63_MOUSE	G T T Y G G G E K R S G G S R I D
tr:Q504P4:Q504P4_MOUSE	G T T Y G G G E K R S G G S R I D
sp:P63017:HSP7C_MOUSE	G T T Y G G G E K R S G G S R I D
sp:O97125:HSP68_DROME	G T T Y G G G E K R S G G S R I D
sp:P29843:HSP7A_DROME	G T T Y G G G E K R S G G S R I D
tr:F3YDI6:F3YDI6_DROME	G T T Y G G G E K R S G G S R I D
sp:P82910:HSP70_DROME	G T T Y G G G E K R S G G S R I D
sp:P02825:HSP71_DROME	G T T Y G G G E K R S G G S R I D
tr:C7LA76:C7LA76_DROME	G T T Y G G G E K R S G G S R I D
sp:P29844:HSP7C_DROME	G T T Y G G G E K R S G G S R I D
sp:P11147:HSP7D_DROME	G T T Y G G G E K R S G G S R I D
tr:C7LA75:C7LA75_DROME	G T T Y G G G E K R S G G S R I D
tr:F3YDH0:F3YDH0_DROME	G T T Y G G G E K R S G G S R I D
tr:F3YDH5:F3YDH5_DROME	G T T Y G G G E K R S G G S R I D
tr:Q8IH88:Q8IH88_DROME	G T T Y G G G E K R S G G S R I D
sp:P09446:HSP7A_CAEEL	G T T Y G G G E K R S G G S R I D
sp:P10592:HSP72_YEAST	G T T Y G G G E K R S G G S R I D

	whole protein		domain-based		contact-based	
protein	ident	simil	ident	simil	ident	simil
PDB:4h5t:chainA:P11142	1.0	1.0	1.0	1.0	1.0	1.0
tr:E7EP94:E7EP94_HUMAN	0.72	0.81	0.77	0.84	1.0	1.0
tr:E9PKE3:E9PKE3_HUMAN	0.97	0.97	0.94	0.94	1.0	1.0
sp:P08107:HSP71_HUMAN	0.84	0.94	0.9	0.97	1.0	1.0
sp:P11142:HSP7C_HUMAN	1.0	1.0	1.0	1.0	1.0	1.0
tr:A8K5I0:A8K5I0_HUMAN	0.84	0.94	0.9	0.97	1.0	1.0
tr:M0R8M9:M0R8M9_RAT	0.99	1.0	0.99	1.0	1.0	1.0
tr:F1LZI1:F1LZI1_RAT	1.0	1.0	0.99	1.0	1.0	1.0
tr:M0RCB1:M0RCB1_RAT	0.98	0.99	0.98	0.98	1.0	1.0
tr:Q3TB63:Q3TB63_MOUSE	0.71	0.71	1.0	1.0	1.0	1.0
tr:Q504P4:Q504P4_MOUSE	0.97	0.97	0.94	0.94	1.0	1.0
sp:P63017:HSP7C_MOUSE	1.0	1.0	1.0	1.0	1.0	1.0
sp:O97125:HSP68_DROME	0.7	0.89	0.84	0.95	1.0	1.0
sp:P29843:HSP7A_DROME	0.8	0.93	0.89	0.97	1.0	1.0
tr:F3YDI6:F3YDI6_DROME	0.73	0.9	0.84	0.95	1.0	1.0
sp:P82910:HSP70_DROME	0.73	0.9	0.84	0.95	1.0	1.0
sp:P02825:HSP71_DROME	0.73	0.9	0.84	0.95	1.0	1.0
tr:C7LA76:C7LA76_DROME	0.73	0.9	0.83	0.95	1.0	1.0
sp:P29844:HSP7C_DROME	0.59	0.83	0.71	0.92	1.0	1.0
sp:P11147:HSP7D_DROME	0.84	0.95	0.92	0.98	1.0	1.0
tr:C7LA75:C7LA75_DROME	0.84	0.95	0.92	0.98	1.0	1.0
tr:F3YDH0:F3YDH0_DROME	0.59	0.83	0.71	0.92	1.0	1.0
tr:F3YDH5:F3YDH5_DROME	0.77	0.9	0.89	0.97	1.0	1.0
tr:Q8IH88:Q8IH88_DROME	0.75	0.88	0.89	0.97	1.0	1.0
sp:P09446:HSP7A_CAEEL	0.84	0.94	0.9	0.97	1.0	1.0
sp:P10592:HSP72_YEAST	0.75	0.92	0.84	0.95	1.0	1.0

Hsp68 (FBgn0001230) associated phenotypes

chemical resistant, long lived, neuroanatomy defective, partially lethal - majority die, some die during pupal stage, starvation stress response defective

(Information from FlyBase)

Hsc70-1 (FBgn0001216) associated phenotypes
partially lethal - majority die, some die during pupal stage

(Information from FlyBase)

Hsc70-1 (UniProt:P29843) annotation
Developmental stage: Heat shock cognate proteins are expressed constitutively during normal development.

(Information from UniProt)

Hsp70Aa (UniProt:P82910) annotation
Induction: Heat shock induces the synthesis of seven proteins at five otherwise inactive sites in the polytene chromosomes of fruit fly larvae. Two separate sites, producing two and three copies, respectively, code for the 70 kDa protein.
Miscellaneous: There are two copies of the gene coding for this protein at chromosome locus 87A7.

(Information from UniProt)

Hsp70Ab (UniProt:P02825) annotation
Induction: Heat shock induces the synthesis of seven proteins at five otherwise inactive sites in the polytene chromosomes of fruit fly larvae. Two separate sites, producing two and three copies, respectively, code for the 70 kDa protein.
Miscellaneous: There are two copies of the gene coding for this protein at chromosome locus 87A7.

(Information from UniProt)

Hsc70-3 (FBgn0001218) associated phenotypes
dominant, neuroanatomy defective, partially lethal - majority die

(Information from FlyBase)

Hsc70-3 (UniProt:P29844) annotation
Function: Probably plays a role in facilitating the assembly of multimeric protein complexes inside the ER.
Subcellular location: Endoplasmic reticulum lumen.

(Information from UniProt)

Hsc70-4 (UniProt:P11147) annotation
Subcellular location: Cytoplasm, perinuclear region. Nucleus. Note=Localized to a meshwork of cytoplasmic fibers around the nucleus. Translocates to the nucleus after thermal stress.
Developmental stage: Heat shock cognate proteins are expressed constitutively during normal development.

(Information from UniProt)

hsp-1 (WBGene00002005) associated phenotypes
lethal, sterile

(Information from WormBase)

DNA topoisomerase 2-alpha

Best gene implication in ageing for this target family came from gene F1M7A0 via mapping the annotation from RGD 62048 annotated in RGD 2014-03-11. Annotation GO 7568 (aging) was Inferred from Expression Pattern

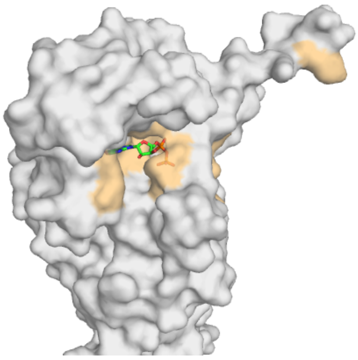

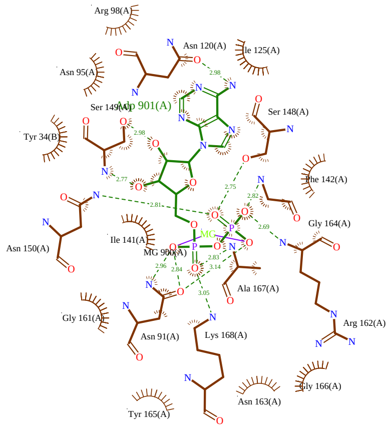

protein	amino acids contacts (binding site)																	
PDB:1zxn:chainA:P11388	Y	N	N	R	N	I	I	F	S	S	N	G	R	N	G	Y	G	A	K
tr:E9PCY5:E9PCY5_HUMAN	Y	N	N	R	N	I	I	F	S	S	N	G	R	N	G	Y	G	A	K
sp:P11388:TOP2A_HUMAN	Y	N	N	R	N	I	I	F	S	S	N	G	R	N	G	Y	G	A	K
sp:Q02880:TOP2B_HUMAN	Y	N	N	R	N	I	I	F	S	S	N	G	R	N	G	Y	G	A	K
tr:F1M7A0:F1M7A0_RAT	Y	N	N	R	N	I	I	F	S	S	N	G	R	N	G	Y	G	A	K
sp:Q01320:TOP2A_MOUSE	Y	N	N	R	N	I	I	F	S	S	N	G	R	N	G	Y	G	A	K
sp:P15348:TOP2_DROME	Y	N	N	R	N	I	I	F	S	S	N	G	R	N	G	Y	G	A	K
sp:Q23670:TOP2_CAEEL	Y	N	N	R	N	I	I	F	S	S	N	G	R	N	G	Y	G	A	K
tr:E9P8H0:E9P8H0_YEASX	Y	N	N	R	N	I	I	F	S	S	N	G	R	N	G	Y	G	A	K
tr:E9P8H2:E9P8H2_YEASX	Y	N	N	R	N	I	I	F	S	S	N	G	R	N	G	Y	G	A	K
tr:E9P8H4:E9P8H4_YEASX	Y	N	N	R	N	I	I	F	S	S	N	G	R	N	G	Y	G	A	K
tr:E9P8H5:E9P8H5_YEASX	Y	N	N	R	N	I	I	F	S	S	N	G	R	N	G	Y	G	A	K
tr:E9P8H7:E9P8H7_YEASX	Y	N	N	R	N	I	I	F	S	S	N	G	R	N	G	Y	G	A	K
tr:E9P8H8:E9P8H8_YEASX	Y	N	N	R	N	I	I	F	S	S	N	G	R	N	G	Y	G	A	K
tr:Q7LMA0:Q7LMA0_YEASX	Y	N	N	R	N	I	I	F	S	S	N	G	R	N	G	Y	G	A	K
tr:E9P8G9:E9P8G9_YEASX	Y	N	N	R	N	I	I	F	S	S	N	G	R	N	G	Y	G	A	K
sp:P06786:TOP2_YEAST	Y	N	N	R	N	I	I	F	S	S	N	G	R	N	G	Y	G	A	K

	whole protein		domain-based		contact-based	
protein	ident	simil	ident	simil	ident	simil
PDB:1zxn:chainA:P11388	0.99	1.0	1.0	1.0	1.0	1.0
tr:E9PCY5:E9PCY5_HUMAN	0.59	0.69	0.73	0.87	1.0	1.0
sp:P11388:TOP2A_HUMAN	1.0	1.0	1.0	1.0	1.0	1.0
sp:Q02880:TOP2B_HUMAN	0.65	0.84	0.72	0.86	1.0	1.0
tr:F1M7A0:F1M7A0_RAT	0.89	0.96	0.96	0.98	1.0	1.0
sp:Q01320:TOP2A_MOUSE	0.89	0.96	0.96	0.99	1.0	1.0
sp:P15348:TOP2_DROME	0.48	0.73	0.65	0.85	1.0	1.0
sp:Q23670:TOP2_CAEEL	0.47	0.73	0.59	0.81	1.0	1.0
tr:E9P8H0:E9P8H0_YEASX	0.36	0.66	0.51	0.78	1.0	1.0
tr:E9P8H2:E9P8H2_YEASX	0.36	0.66	0.51	0.78	1.0	1.0
tr:E9P8H4:E9P8H4_YEASX	0.36	0.66	0.51	0.78	1.0	1.0
tr:E9P8H5:E9P8H5_YEASX	0.36	0.66	0.51	0.78	1.0	1.0
tr:E9P8H7:E9P8H7_YEASX	0.36	0.66	0.51	0.78	1.0	1.0
tr:E9P8H8:E9P8H8_YEASX	0.36	0.66	0.51	0.78	1.0	1.0
tr:Q7LMA0:Q7LMA0_YEASX	0.36	0.66	0.51	0.78	1.0	1.0
tr:E9P8G9:E9P8G9_YEASX	0.36	0.66	0.51	0.78	1.0	1.0
sp:P06786:TOP2_YEAST	0.36	0.66	0.51	0.78	1.0	1.0

Top2 (FBgn0003732) associated phenotypes

developmental rate defective, heat sensitive, lethal - all die before end of first instar larval stage, lethal - all die before end of second instar larval stage, lethal - all die during first instar larval stage, male fertile, neuroanatomy defective, partially lethal - majority die, small body, somatic clone

(Information from FlyBase)

Top2 (UniProt:P15348) annotation

Function: Control of topological states of DNA by transient breakage and subsequent rejoining of DNA strands. Topoisomerase II makes double-strand breaks.

Cofactor: Mg(2+) Evidence=(PROSITE-ProRule:PRU00995); Name=Mn(2+); Xref=ChEBI:CHEBI:29035; Evidence=(PROSITE-ProRule:PRU00995); Name=Ca(2+); Xref=ChEBI:CHEBI:29108; Evidence=(PROSITE-ProRule:PRU00995); Note=Binds two Mg(2+) per subunit. The magnesium ions form salt bridges with both the protein and the DNA. Can also accept other divalent metal cations, such as Mn(2+) or Ca(2+). (PROSITE-ProRule:PRU00995);

Subunit: Homodimer.

Subcellular location: Nucleus.

Miscellaneous: Eukaryotic topoisomerase I and II can relax both negative and positive supercoils, whereas prokaryotic enzymes relax only negative supercoils.

(Information from UniProt)

K12D12.1 (UniProt:Q23670) annotation

Function: Control of topological states of DNA by transient breakage and subsequent rejoining of DNA strands. Topoisomerase II makes double-strand breaks (By similarity).

Cofactor: Mg(2+) Evidence=(PROSITE-ProRule:PRU00995); Name=Mn(2+); Xref=ChEBI:CHEBI:29035; Evidence=(PROSITE-ProRule:PRU00995); Name=Ca(2+); Xref=ChEBI:CHEBI:29108; Evidence=(PROSITE-ProRule:PRU00995); Note=Binds two Mg(2+) per subunit. The magnesium ions form salt bridges with both the protein and the DNA. Can also accept other divalent metal cations, such as Mn(2+) or Ca(2+). (PROSITE-ProRule:PRU00995);

Subunit: Homodimer.

Subcellular location: Nucleus

Miscellaneous: Eukaryotic topoisomerase I and II can relax both negative and positive supercoils, whereas prokaryotic enzymes relax only negative supercoils.

(Information from UniProt)

5'-AMP-activated protein kinase subunit gamma

Best gene implication in ageing for this target family came from gene P12904 annotated in UniProt release 2014_02. Annotation GO subterm of 7568 (aging) was Inferred from Mutant Phenotype

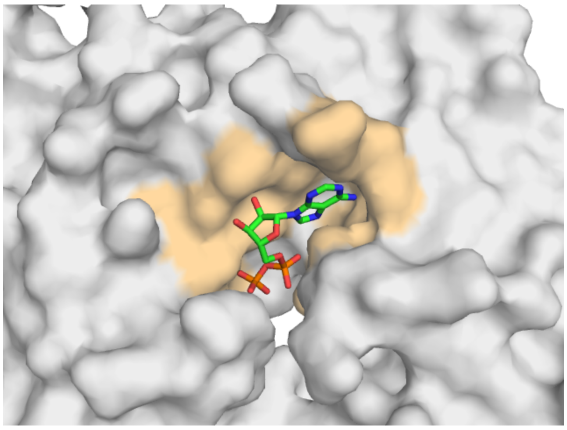

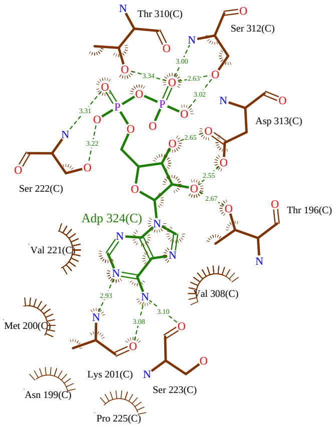

protein	amino acids contacts (binding site)													
PDB:3t4n:chainC:P12904	Q	T	N	M	K	V	S	S	P	V	T	L	S	D
tr:F8W9D1:F8W9D1_HUMAN	H	T	N	I	A	V	S	A	P	I	S	L	S	D
tr:F8VYY9:F8VYY9_HUMAN	H	T	N	I	A	V	S	A	P	I	S	L	S	D
tr:B4DT70:B4DT70_HUMAN	H	T	D	L	A	V	S	A	P	V	S	L	S	D
sp:P54619:AAKG1_HUMAN	H	T	N	I	A	V	S	A	P	I	S	L	S	D
sp:Q9UGI9:AAKG3_HUMAN	H	T	D	L	A	V	S	A	P	V	S	L	S	D
sp:P80385:AAKG1_RAT	H	T	N	I	A	V	S	A	P	I	S	L	S	D
tr:D4A7E4:D4A7E4_RAT	H	T	D	L	A	V	S	A	P	V	S	L	S	D
sp:O54950:AAKG1_MOUSE	H	T	N	I	A	V	S	A	P	I	S	L	S	D
tr:Q3TWR3:Q3TWR3_MOUSE	H	T	N	I	A	V	S	A	P	I	S	L	S	D
sp:Q8BGM7:AAKG3_MOUSE	H	T	D	L	A	V	S	A	P	V	S	L	S	D
tr:Q7KNS9:Q7KNS9_DROME	H	T	N	I	E	V	S	A	P	I	S	L	S	D
tr:Q8IGX0:Q8IGX0_DROME	H	T	N	I	E	V	S	A	P	I	S	L	S	D
tr:Q0KI34:Q0KI34_DROME	H	T	N	I	E	V	S	A	P	I	S	L	S	D
tr:A1A703:A1A703_DROME	H	T	N	I	E	V	S	A	P	I	S	L	S	D
tr:A1A6Y3:A1A6Y3_DROME	H	T	N	I	E	V	S	A	P	I	S	L	S	D
tr:O96613:O96613_DROME	H	T	N	I	E	V	S	A	P	I	S	L	S	D
tr:Q8I0K6:Q8I0K6_DROME	H	T	N	I	E	V	S	A	P	I	S	L	S	D
tr:Q8I0A1:Q8I0A1_DROME	H	T	N	I	E	V	S	A	P	I	S	L	S	D
tr:Q8SXT8:Q8SXT8_DROME	H	T	N	I	E	V	S	A	P	I	S	L	S	D
tr:Q8I0B2:Q8I0B2_DROME	H	T	N	I	E	V	S	A	P	I	S	L	S	D
tr:Q7JQX9:Q7JQX9_DROME	H	T	N	I	E	V	S	A	P	I	S	L	S	D
tr:Q9VDD2:Q9VDD2_DROME	H	T	N	I	E	V	S	A	P	I	S	L	S	D
tr:Q9BHL6:Q9BHL6_CAEEL	H	A	D	I	L	V	S	A	P	V	S	L	S	D
sp:P12904:AAKG_YEAST	Q	T	N	M	K	V	S	S	P	V	T	L	S	D
tr:F4ZZV2:F4ZZV2_YEASX	Q	T	N	M	K	V	S	S	P	V	T	L	S	D

	whole protein		domain-based		contact-based	
protein	ident	simil	ident	simil	ident	simil
PDB:3t4n:chainC:P12904	1.0	1.0	1.0	1.0	1.0	1.0
tr:F8W9D1:F8W9D1_HUMAN	0.23	0.56	0.24	0.57	0.57	0.78
tr:F8VYY9:F8VYY9_HUMAN	0.28	0.63	0.29	0.65	0.57	0.78
tr:B4DT70:B4DT70_HUMAN	0.3	0.69	0.31	0.71	0.57	0.78
sp:P54619:AAKG1_HUMAN	0.3	0.71	0.32	0.75	0.57	0.78
sp:Q9UGI9:AAKG3_HUMAN	0.2	0.49	0.31	0.74	0.57	0.78
sp:P80385:AAKG1_RAT	0.3	0.72	0.32	0.75	0.57	0.78
tr:D4A7E4:D4A7E4_RAT	0.2	0.49	0.31	0.75	0.57	0.78
sp:O54950:AAKG1_MOUSE	0.3	0.72	0.32	0.75	0.57	0.78
tr:Q3TWR3:Q3TWR3_MOUSE	0.3	0.72	0.32	0.75	0.57	0.78
sp:Q8BGM7:AAKG3_MOUSE	0.2	0.49	0.31	0.75	0.57	0.78
tr:Q7KNS9:Q7KNS9_DROME	0.21	0.47	0.33	0.73	0.57	0.82
tr:Q8IGX0:Q8IGX0_DROME	0.19	0.45	0.33	0.75	0.57	0.82
tr:Q0KI34:Q0KI34_DROME	0.2	0.45	0.33	0.75	0.57	0.82
tr:A1A703:A1A703_DROME	0.17	0.39	0.33	0.75	0.57	0.82
tr:A1A6Y3:A1A6Y3_DROME	0.17	0.39	0.33	0.75	0.57	0.82
tr:O96613:O96613_DROME	0.16	0.37	0.33	0.75	0.57	0.82
tr:Q8I0K6:Q8I0K6_DROME	0.15	0.34	0.33	0.75	0.57	0.82
tr:Q8I0A1:Q8I0A1_DROME	0.13	0.3	0.33	0.75	0.57	0.82
tr:Q8SXT8:Q8SXT8_DROME	0.12	0.27	0.33	0.75	0.57	0.82
tr:Q8I0B2:Q8I0B2_DROME	0.12	0.27	0.33	0.75	0.57	0.82
tr:Q7JQX9:Q7JQX9_DROME	0.11	0.26	0.33	0.75	0.57	0.82
tr:Q9VDD2:Q9VDD2_DROME	0.08	0.17	0.33	0.75	0.57	0.82
tr:Q9BHL6:Q9BHL6_CAEEL	0.17	0.41	0.32	0.74	0.5	0.75
sp:P12904:AAKG_YEAST	1.0	1.0	1.0	1.0	1.0	1.0
tr:F4ZZV2:F4ZZV2_YEASX	1.0	1.0	1.0	1.0	1.0	1.0

SNF4Agamma (FBgn0264357) associated phenotypes

feeding behavior defective, hyperactive, neuroanatomy defective, nutrition conditional, phototaxis defective, short lived, starvation stress response defective

(Information from FlyBase)

Mitogen-activated protein kinase FUS3

Best gene implication in ageing for this target family came from gene P39745 via mapping the annotation from WormBase WBGene00003401 annotated in WormBase 2014-03-11. Annotation GO 8340 (determination of adult lifespan) was Inferred from Mutant Phenotype

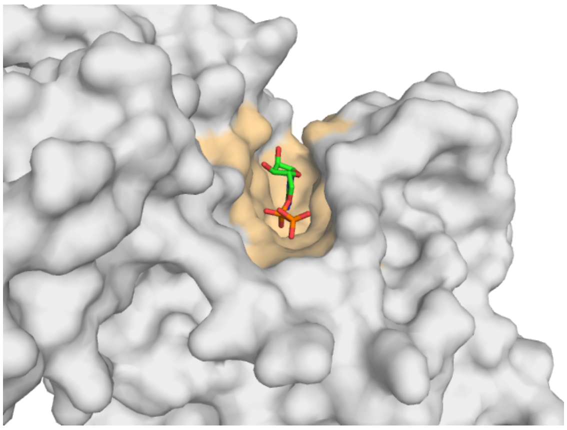

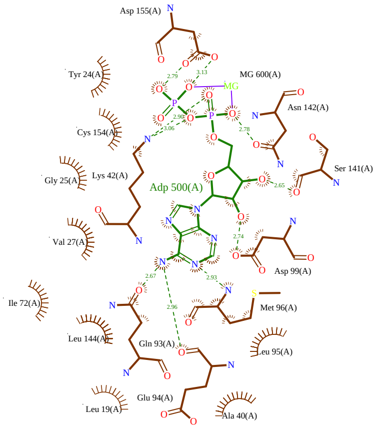

protein	amino acids contacts (binding site)
PDB:2b9f:chainA:P16892	L Y G V A K R I Q E L M D R S N L C D
PDB:2b9h:chainA:P16892	L Y G V A K R I Q E L M D R S N L C D
PDB:2b9i:chainA:P16892	L Y G V A K R I Q E L M D R S N L C D
PDB:2b9j:chainA:P16892	L Y G V A K R I Q E L M D R S N L C D
PDB:2f9g:chainA:P16892	L Y G V A K R I Q E L M D R S N L C D
tr:E9PJF0:E9PJF0_HUMAN	I Y G V A K R I Q D L M D K S N L C D
tr:E9PQW4:E9PQW4_HUMAN	I Y G V A K R I Q D L M D K S N L C D
sp:P27361:MK03_HUMAN	I Y G V A K R I Q D L M D K S N L C D
tr:L7RXH5:L7RXH5_HUMAN	I Y G V A K R I Q D L M D K S N L C D
sp:P21708:MK03_RAT	I Y G V A K R I Q D L M D K S N L C D
sp:Q63844:MK03_MOUSE	I Y G V A K R I Q D L M D K S N L C D
tr:D3Z3G6:D3Z3G6_MOUSE	I Y G V A K R I Q D L M D K S N L C D
sp:P40417:ERKA_DROME	I Y G V A K R I Q C L M D K S N L C D
tr:D9PTS4:D9PTS4_DROME	I Y G V A K R I Q C L M D K S N L C D
sp:P39745:SUR1_CAEEL	I Y G V A K R I Q C L M D K S N L C D
sp:P16892:FUS3_YEAST	L Y G V A K R I Q E L M D R S N L C D

	whole protein		domain-based		contact-based	
protein	ident	simil	ident	simil	ident	simil
PDB:2b9f:chainA:P16892	1.0	1.0	1.0	1.0	1.0	1.0
PDB:2b9h:chainA:P16892	0.99	1.0	0.99	1.0	1.0	1.0
PDB:2b9i:chainA:P16892	0.99	1.0	0.99	1.0	1.0	1.0
PDB:2b9j:chainA:P16892	0.99	1.0	0.99	1.0	1.0	1.0
PDB:2f9g:chainA:P16892	1.0	1.0	1.0	1.0	1.0	1.0
tr:E9PJF0:E9PJF0_HUMAN	0.38	0.63	0.47	0.77	0.84	0.96
tr:E9PQW4:E9PQW4_HUMAN	0.41	0.68	0.5	0.8	0.84	0.96
sp:P27361:MK03_HUMAN	0.45	0.74	0.53	0.84	0.84	0.96
tr:L7RXH5:L7RXH5_HUMAN	0.45	0.74	0.53	0.84	0.84	0.96
sp:P21708:MK03_RAT	0.45	0.74	0.53	0.84	0.84	0.96
sp:Q63844:MK03_MOUSE	0.45	0.74	0.53	0.84	0.84	0.96
tr:D3Z3G6:D3Z3G6_MOUSE	0.41	0.69	0.53	0.84	0.84	0.96
sp:P40417:ERKA_DROME	0.45	0.75	0.54	0.84	0.84	0.9
tr:D9PTS4:D9PTS4_DROME	0.42	0.69	0.54	0.84	0.84	0.9
sp:P39745:SUR1_CAEEL	0.38	0.63	0.52	0.83	0.84	0.9
sp:P16892:FUS3_YEAST	1.0	1.0	1.0	1.0	1.0	1.0

rl (FBgn0003256) associated phenotypes

cell lethal, chemical resistant, chemical sensitive, decreased cell size, developmental rate defective,

dominant, increased cell number, large body, lethal - all die before end of pupal stage, maternal effect, mitotic cell cycle defective, neuroanatomy defective, neurophysiology defective, partially, partially lethal - majority die, radiation sensitive, short lived, size defective, somatic clone, some die during pupal stage, visual behavior defective, wound healing defective

(Information from FlyBase)

rl (UniProt:P40417) annotation

Function: Required downstream of Raf in the sevenless (sev), torso (tor), and Drosophila EGF receptor homolog (DER) signal transduction pathways. Required for embryonic epithelial tissue repair. (PubMed:22140578, PubMed:8157002).
Cofactor:Mg(2+)
Enzyme regulation: Activated by tyrosine and threonine phosphorylation.
Subcellular location: Cytoplasm ECO:0000269—PubMed:1378625. Nucleus (PubMed:1378625).
Tissue specificity: In third instar larvae, expressed in eye imaginal disks. In adults, expressed in head and body. (PubMed:1378625).
Developmental stage: Embryos, larvae and adults. (PubMed:1378625).
Domain: The TXY motif contains the threonine and tyrosine residues whose phosphorylation activates the MAP kinases.
Ptm: Dually phosphorylated on Thr-198 and Tyr-200, which activates the enzyme (By similarity). Phosphorylated on tyrosine residue(s) in response to insulin. (, PubMed:1378625, PubMed:18327897).
Disruption phenotype: Embryonic wound healing defects. (PubMed:22140578).

(Information from UniProt)

rl-RB (UniProt:D9PTS4) annotation

Cofactor: Mg(2+) Evidence=(RuleBase:RU361165);
Enzyme regulation: Activated by threonine and tyrosine phosphorylation. (RuleBase:RU361165).

(Information from UniProt)

mpk-1 (WBGene00003401) associated phenotypes

P lineages variant, antibody staining variant, bacterially unswollen, cell corpse number variant, constipated, egg laying variant, exaggerated asynchrony early emb, germ cell arrest, germ nuclei rachis, lineage variant, metabolic pathway variant, oocytes disorganized, pachytene arrested germline nuclei, pachytene progression during oogenesis variant, pachytene region organization variant, pathogen susceptibility increased, protein subcellular localization variant, rod like larval lethal, sterile, vulvaless

(Information from WormBase)

mpk-1 (UniProt:P39745) annotation

Function: Function in let-60 Ras signaling pathway; acts downstream of lin-45 raf kinase, but before the lin-1 gene product in controlling vulval cell differentiation (PubMed:8299935, PubMed:8299936). In oocytes, inhibits the activity of the chloride channel clh-3, likely by activating gck-3 (PubMed:21160027). (PubMed:21160027, PubMed:8299935, PubMed:8299936).
Cofactor:Mg(2+)
Enzyme regulation: Activated by tyrosine and threonine phosphorylation.
Domain: The TXY motif contains the threonine and tyrosine residues whose phosphorylation activates the MAP kinases.
Ptm: Dually phosphorylated on Thr-256 and Tyr-258, which activates the enzyme.

(Information from UniProt)

Glutamate–cysteine ligase

Best gene implication in ageing for this target family came from gene A4V448 via mapping the annotation from Ensembl FBgn0040319 via mapping the annotation from EntrezGene 53581 via mapping the annotation from GenAgeModels 0293 annotated in GenAge release 17.

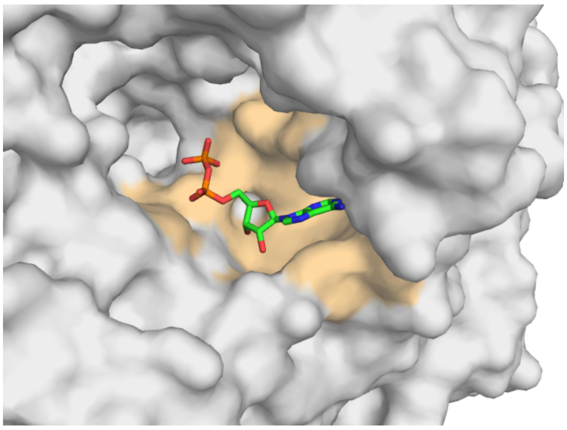

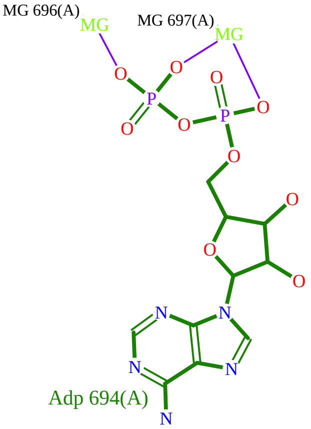

protein	amino acids contacts (binding site)
PDB:3ig8:chainA:P32477	F W G D E H E T P P T F Q K P R E
PDB:3lvv:chainA:P32477	F W G D E H E T P P T F Q K P R E
sp:P48506:GSH1_HUMAN	K W G D E R E T P P T F Q K I R E
tr:Q14TF0:Q14TF0_HUMAN	K W G D E R E T P P T F Q K I R E
sp:P19468:GSH1_RAT	K W G D E R E T P P T F Q K I R E
tr:Q3TEF1:Q3TEF1_MOUSE	K W G D E R E T P P T F Q K I R E
sp:P97494:GSH1_MOUSE	K W G D E R E T P P T F Q K I R E
tr:A4V448:A4V448_DROME	K W G D E R E T P P T F Q K I R E
sp:Q9W3K5:GSH1_DROME	K W G D E R E T P P T F Q K I R E
sp:Q20117:GSH1_CAEEL	K W G D E R E T P P T F Q K I R E
tr:E9P9G0:E9P9G0_YEASX	F W G D E H E T P P T F Q K P R E
tr:E9P9M0:E9P9M0_YEASX	F W G D E H E T P P T F Q K P R E
tr:F5CIK1:F5CIK1_YEASX	F W G D E H E T P P T F Q K P R E
sp:P32477:GSH1_YEAST	F W G D E H E T P P T F Q K P R E

	whole protein		domain-based		contact-based	
protein	ident	simil	ident	simil	ident	simil
PDB:3ig8:chainA:P32477	1.0	1.0	1.0	1.0	1.0	1.0
PDB:3lvv:chainA:P32477	1.0	1.0	1.0	1.0	1.0	1.0
sp:P48506:GSH1_HUMAN	0.4	0.72	0.41	0.72	0.82	0.87
tr:Q14TF0:Q14TF0_HUMAN	0.4	0.72	0.41	0.72	0.82	0.87
sp:P19468:GSH1_RAT	0.41	0.72	0.41	0.72	0.82	0.87
tr:Q3TEF1:Q3TEF1_MOUSE	0.41	0.72	0.41	0.72	0.82	0.87
sp:P97494:GSH1_MOUSE	0.41	0.72	0.41	0.72	0.82	0.87
tr:A4V448:A4V448_DROME	0.33	0.62	0.36	0.63	0.82	0.87
sp:Q9W3K5:GSH1_DROME	0.33	0.62	0.36	0.63	0.82	0.87
sp:Q20117:GSH1_CAEEL	0.34	0.68	0.37	0.7	0.82	0.87
tr:E9P9G0:E9P9G0_YEASX	1.0	1.0	1.0	1.0	1.0	1.0
tr:E9P9M0:E9P9M0_YEASX	1.0	1.0	1.0	1.0	1.0	1.0
tr:F5CIK1:F5CIK1_YEASX	1.0	1.0	0.99	1.0	1.0	1.0
sp:P32477:GSH1_YEAST	1.0	1.0	1.0	1.0	1.0	1.0

Gclc (FBgn0040319) associated phenotypes

chemical resistant, long lived

(Information from FlyBase)

Gclc (UniProt:Q9W3K5) annotation

Pathway: Sulfur metabolism; glutathione biosynthesis; glutathione from L-cysteine and L-glutamate:

step 1/2.

(Information from UniProt)

gcs-1 (UniProt:Q20117) annotation

Pathway: Sulfur metabolism; glutathione biosynthesis; glutathione from L-cysteine and L-glutamate: step 1/2.

(Information from UniProt)

Heat shock 70 kDa protein 1-like

Best gene implication in ageing for this target family came from gene O97125 annotated in UniProt release 2014_02. Annotation GO 8340 (determination of adult lifespan) was Inferred from Mutant Phenotype

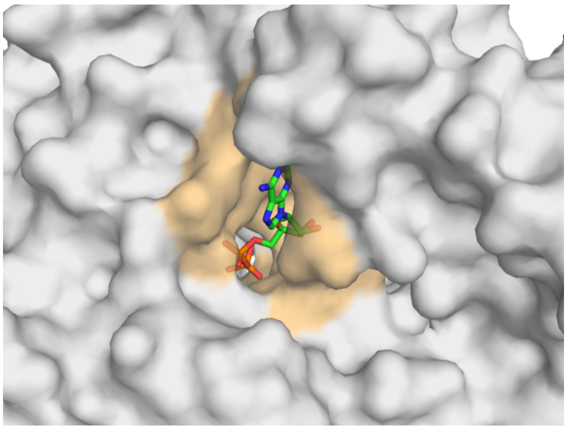
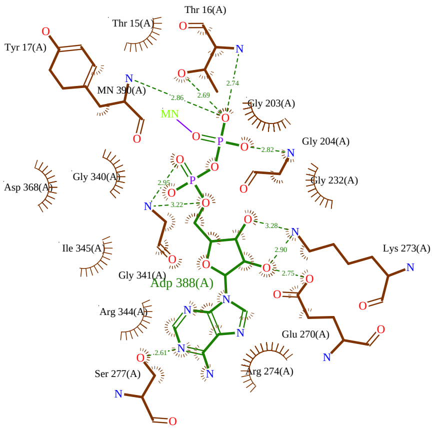

protein	amino acids contacts (binding site)
PDB:3gdq:chainA:P34931	T T Y G G G E K R S G G S R I D
sp:P34931:HS71L_HUMAN	T T Y G G G E K R S G G S R I D
tr:M0R8M9:M0R8M9_RAT	T T Y G G G E K R S G G S R I D
sp:P17879:HS71B_MOUSE	T T Y G G G E K R S G G S R I D
sp:O97125:HSP68_DROME	T T Y G G G E K R S G G S R I D
tr:C7LA76:C7LA76_DROME	T T Y G G G E K R S G G S R I D
sp:P82910:HSP70_DROME	T T Y G G G E K R S G G S R I D
sp:P02825:HSP71_DROME	T T Y G G G E K R S G G S R I D
tr:F3YDI6:F3YDI6_DROME	T T Y G G G E K R S G G S R I D
sp:P09446:HSP7A_CAEEL	T T Y G G G E K R S G G S R I D
sp:P10592:HSP72_YEAST	T T Y G G G E K R S G G S R I D

	whole protein		domain-based		contact-based	
protein	ident	simil	ident	simil	ident	simil
PDB:3gdq:chainA:P34931	1.0	1.0	1.0	1.0	1.0	1.0
sp:P34931:HS71L_HUMAN	1.0	1.0	1.0	1.0	1.0	1.0
tr:M0R8M9:M0R8M9_RAT	0.81	0.93	0.88	0.97	1.0	1.0
sp:P17879:HS71B_MOUSE	0.87	0.95	0.96	0.99	1.0	1.0
sp:O97125:HSP68_DROME	0.71	0.89	0.84	0.95	1.0	1.0
tr:C7LA76:C7LA76_DROME	0.73	0.9	0.84	0.95	1.0	1.0
sp:P82910:HSP70_DROME	0.73	0.9	0.84	0.95	1.0	1.0
sp:P02825:HSP71_DROME	0.73	0.9	0.84	0.95	1.0	1.0
tr:F3YDI6:F3YDI6_DROME	0.73	0.9	0.84	0.95	1.0	1.0
sp:P09446:HSP7A_CAEEL	0.8	0.93	0.87	0.96	1.0	1.0
sp:P10592:HSP72_YEAST	0.72	0.9	0.82	0.95	1.0	1.0

Hsp68 (FBgn0001230) associated phenotypes

chemical resistant, long lived, neuroanatomy defective, partially lethal - majority die, some die during pupal stage, starvation stress response defective

(Information from FlyBase)

Hsp70Aa (UniProt:P82910) annotation
Induction: Heat shock induces the synthesis of seven proteins at five otherwise inactive sites in the polytene chromosomes of fruit fly larvae. Two separate sites, producing two and three copies, respectively, code for the 70 kDa protein.
Miscellaneous: There are two copies of the gene coding for this protein at chromosome locus 87A7.

(Information from UniProt)

Hsp70Ab (UniProt:P02825) annotation
Induction: Heat shock induces the synthesis of seven proteins at five otherwise inactive sites in the polytene chromosomes of fruit fly larvae. Two separate sites, producing two and three copies, respectively, code for the 70 kDa protein.
Miscellaneous: There are two copies of the gene coding for this protein at chromosome locus 87A7.

(Information from UniProt)

hsp-1 (WBGene00002005) associated phenotypes
lethal, sterile

(Information from WormBase)

DNA mismatch repair protein Mlh1

Best gene implication in ageing for this target family came from gene P40692 via mapping the annotation from Ensembl ENSG00000076242 via mapping the annotation from EntrezGene 4292 via mapping the annotation from GenAgeHuman 0243 annotated in GenAge release 17.

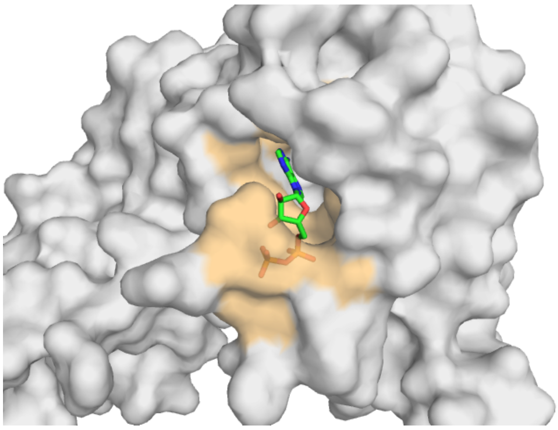

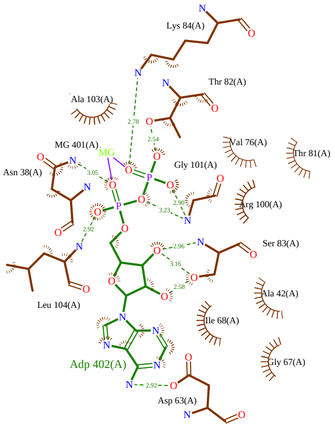

protein	amino acids contacts (binding site)														
PDB:4p7a:chainA:P40692	N	A	D	G	I	V	T	T	S	K	R	G	A	L	T
sp:P40692:MLH1_HUMAN	N	A	D	G	I	V	T	T	S	K	R	G	A	L	T
tr:Q5GJ64:Q5GJ64_HUMAN	N	A	D	G	I	V	T	T	S	K	R	G	A	L	T
tr:F1LSD8:F1LSD8_RAT	N	A	D	G	I	V	T	T	S	K	R	G	A	L	T
sp:Q9JK91:MLH1_MOUSE	N	A	D	G	I	V	T	T	S	K	R	G	A	L	T
tr:A1Z7C1:A1Z7C1_DROME	N	A	D	G	I	V	T	T	S	K	R	G	A	L	T
tr:O61917:O61917_DROME	N	A	D	G	I	V	T	T	S	K	R	G	A	L	T
tr:Q9XU10:Q9XU10_CAEEL	N	A	D	G	I	V	A	T	S	K	R	G	A	L	T
sp:P38920:MLH1_YEAST	N	A	D	G	I	L	T	T	S	K	R	G	A	L	T

protein	whole protein ident	whole protein simil	domain-based ident	domain-based simil	contact-based ident	contact-based simil
PDB:4p7a:chainA:P40692	1.0	1.0	1.0	1.0	1.0	1.0
sp:P40692:MLH1_HUMAN	1.0	1.0	1.0	1.0	1.0	1.0
tr:Q5GJ64:Q5GJ64_HUMAN	1.0	1.0	1.0	1.0	1.0	1.0
tr:F1LSD8:F1LSD8_RAT	0.88	0.96	0.96	0.99	1.0	1.0
sp:Q9JK91:MLH1_MOUSE	0.88	0.96	0.95	0.99	1.0	1.0
tr:A1Z7C1:A1Z7C1_DROME	0.45	0.73	0.68	0.91	1.0	1.0
tr:O61917:O61917_DROME	0.44	0.72	0.68	0.91	1.0	1.0
tr:Q9XU10:Q9XU10_CAEEL	0.3	0.67	0.54	0.85	0.93	0.96
sp:P38920:MLH1_YEAST	0.36	0.72	0.6	0.87	0.93	0.98

Serine/threonine-protein kinase mTOR

Best gene implication in ageing for this target family came from gene Q9VK45 annotated in UniProt release 2014_02. Annotation GO 8340 (determination of adult lifespan) was Inferred from Mutant Phenotype

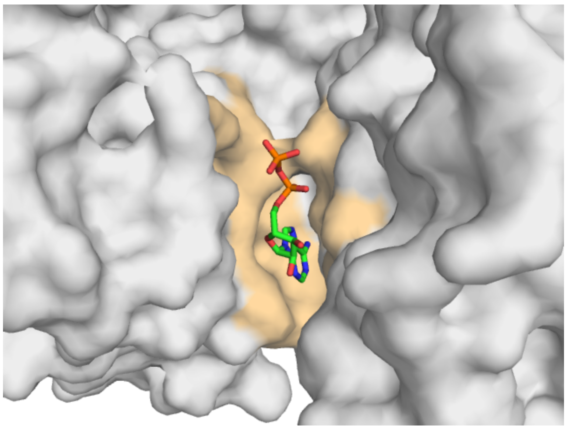

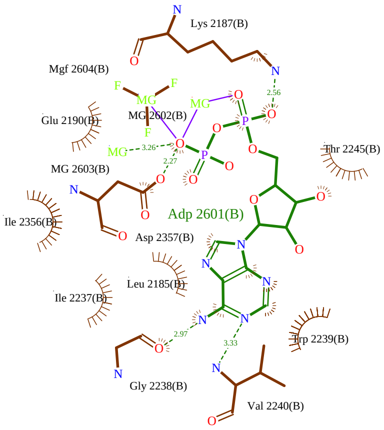

protein	amino acids contacts (binding site)											
PDB:4jsv:chainB:P42345	Q	L	K	E	I	G	W	V	T	S	N	I	D
tr:B1AKP8:B1AKP8_HUMAN	Q	L	K	E	I	G	W	V	T	S	N	I	D
sp:P42345:MTOR_HUMAN	Q	L	K	E	I	G	W	V	T	S	N	I	D
sp:P42346:MTOR_RAT	Q	L	K	E	I	G	W	V	T	S	N	I	D
sp:Q9JLN9:MTOR_MOUSE	Q	L	K	E	I	G	W	V	T	S	N	I	D
tr:Q8T9I6:Q8T9I6_DROME	Q	L	K	E	I	G	W	V	T	S	N	I	D
tr:Q960H0:Q960H0_DROME	Q	L	K	E	I	G	W	V	T	S	N	I	D
tr:H1ZYC1:H1ZYC1_DROME	Q	L	K	E	I	G	W	V	T	S	N	I	D
sp:Q9VK45:TOR_DROME	Q	L	K	E	I	G	W	V	T	S	N	I	D
tr:H1ZYB7:H1ZYB7_DROME	Q	L	K	E	I	G	W	V	T	S	N	I	D
tr:H1ZYB5:H1ZYB5_DROME	Q	L	K	E	I	G	W	V	T	S	N	I	D
sp:Q95Q95:TOR_CAEEL	Q	L	K	E	I	G	W	V	T	S	N	I	D
sp:P32600:TOR2_YEAST	Q	V	K	E	L	G	W	V	T	S	N	I	D

	whole protein		domain-based		contact-based	
protein	ident	simil	ident	simil	ident	simil
PDB:4jsv:chainB:P42345	1.0	1.0	1.0	1.0	1.0	1.0
tr:B1AKP8:B1AKP8_HUMAN	0.3	0.3	1.0	1.0	1.0	1.0
sp:P42345:MTOR_HUMAN	1.0	1.0	1.0	1.0	1.0	1.0
sp:P42346:MTOR_RAT	0.99	1.0	0.99	1.0	1.0	1.0
sp:Q9JLN9:MTOR_MOUSE	0.99	1.0	1.0	1.0	1.0	1.0
tr:Q8T9I6:Q8T9I6_DROME	0.13	0.16	0.78	0.93	1.0	1.0
tr:Q960H0:Q960H0_DROME	0.2	0.26	0.78	0.93	1.0	1.0
tr:H1ZYC1:H1ZYC1_DROME	0.52	0.81	0.78	0.93	1.0	1.0
sp:Q9VK45:TOR_DROME	0.52	0.81	0.78	0.93	1.0	1.0
tr:H1ZYB7:H1ZYB7_DROME	0.52	0.81	0.78	0.93	1.0	1.0
tr:H1ZYB5:H1ZYB5_DROME	0.52	0.81	0.78	0.93	1.0	1.0
sp:Q95Q95:TOR_CAEEL	0.31	0.66	0.65	0.88	1.0	1.0
sp:P32600:TOR2_YEAST	0.39	0.71	0.65	0.89	0.85	0.97

Tor (FBgn0021796) associated phenotypes

body size defective, cell cycle defective, cell size defective, chemical sensitive, decreased cell number, decreased cell size, developmental rate defective, dominant, germline clone, gravitaxis defective, increased cell death, increased cell size, lethal - all die before end of pupal stage, lethal - all die before end of second instar larval stage, long lived, mitotic cell cycle defective, neuroanatomy defective, nutrition conditional, size defective, small body, somatic clone, some die during pupal stage, some die during second instar larval stage, some die during third instar larval stage, sterile

(Information from FlyBase)

Tor (UniProt:Q9VK45) annotation

Function: Promotes cell and tissue growth, maintains tissue homeostatis and controls responses to environmental stress and aging. Regulates growth during animal development by coupling growth factor signaling to nutrient availability. Central regulators of autophagy. May be involved in atg1 phosphorylation. May also be involved, directly or indirectly, in the control of neuronal function. Phosphorylates S6K/p70S6K, in vitro. May regulate the activity of S6K. Overexpression inhibits growth and reduces cell size. Affects the timing of neuronal cell differentiation. Hyperactivation of the signaling leads to accelerated differentiation, whereas inhibition of the signaling retards differentiation. Thus, in addition to controlling growth of the cell in which it resides, it can also influence growth of distant cells and organs during development via a humoral mechanism. In short, during development, it primarily controls growth, whereas in the adult, where there is relatively little growth, it controls aging and other aspects of nutrient-related physiology. Rag GTPases act as activators of TORC1 in response to amino acid signals. (PubMed:11069885, PubMed:11069888, PubMed:12559758, PubMed:14505573, PubMed:15454083, PubMed:16469695, PubMed:18604198, PubMed:19211682, PubMed:19225150).

Subunit: May be part of a minimal complex, TORC1, consisting oftor, raptor and lst8. May be part of a minimal complex, TORC2, consisting of tor, rictor and lst8 (By similarity). Self-associates; assembles into homomultimeric complexes. Component of a multiprotein complex. (, PubMed:16219781).

Induction: By PI3K/Akt signaling, or by nutrients such as amino acids, and by high cellular energy levels. (PubMed:19211682).

Disruption phenotype: Not lethal. Displays phenotypes characteristic of amino acid deprivation, including reduced nucleolar size, lipid vesicle aggregation in the larval fat body, and a cell type-specific pattern of cell cycle arrest that can be bypassed by overexpression of the S-phase regulator cyclin E. Reach only the size of second instar larvae, at which point they undergo cell cycle arrest. (PubMed:11069885, PubMed:11069888).

(Information from UniProt)

let-363 (WBGene00002583) associated phenotypes

adult growth variant, late larval lethal, lethal

(Information from WormBase)

let-363 (UniProt:Q95Q95) annotation
Function: Kinase that regulates the mRNA translation machinery, probably by modulating the activity of translation factors such as eIF-4G and eIF-2. It may have some protein kinase activity instead of lipid kinase activity. May act as a mediator of lifespan regulation by insulin signaling and nutrient sensing. (PubMed:12225660, PubMed:14668850).
Subcellular location: Nucleus (PubMed:12225660).
Tissue specificity: Ubiquitous. Expressed in all major tissues and organs, including the intestine, gonads and hypodermal cells. (PubMed:12225660).
Disruption phenotype: Defects strongly increase lifespan, the mean lifetime being of 25 days instead of 10 days, suggesting that it may be involved in aging process. (PubMed:14668850).

(Information from UniProt)

DNA mismatch repair protein Msh2

Best gene implication in ageing for this target family came from gene Q3TZI5 annotated in UniProt release 2014_02. Annotation GO 8340 (determination of adult lifespan) was Inferred from Mutant Phenotype

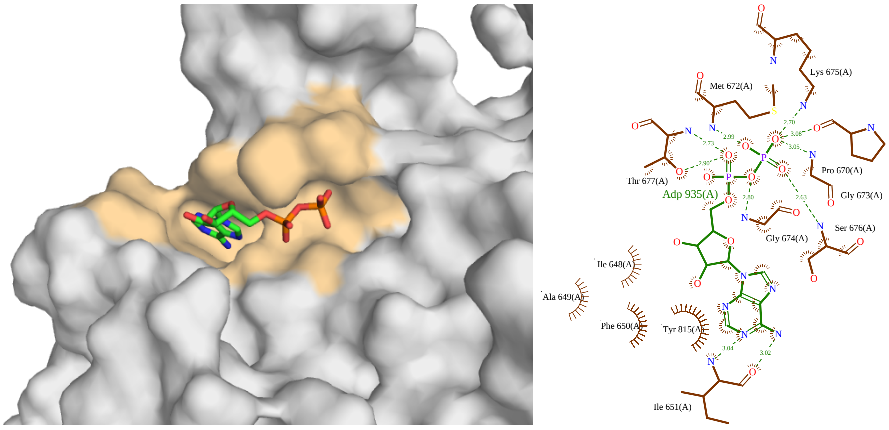

protein	amino acids contacts (binding site)
PDB:2o8b:chainA:P43246	V Q I A F I N P N M G G K S T Y
PDB:2o8c:chainA:P43246	V Q I A F I N P N M G G K S T Y
PDB:2o8d:chainA:P43246	V Q I A F I N P N M G G K S T Y
PDB:2o8e:chainA:P43246	V Q I A F I N P N M G G K S T Y
PDB:2o8f:chainA:P43246	V Q I A F I N P N M G G K S T Y
PDB:3thw:chainA:P43246	V Q I A F I N P N M G G K S T Y
PDB:3thx:chainA:P43246	V Q I A F I N P N M G G K S T Y
PDB:3thy:chainA:P43246	V Q I A F I N P N M G G K S T Y
PDB:3thz:chainA:P43246	V Q I A F I N P N M G G K S T Y
tr:B4DL39:B4DL39_HUMAN	V Q I A F I N P N M G G K S T Y
tr:E9PHA6:E9PHA6_HUMAN	V Q I A F I N P N M G G K S T Y
sp:P43246:MSH2_HUMAN	V Q I A F I N P N M G G K S T Y
tr:B1WBQ7:B1WBQ7_RAT	V Q V A F I N P N M G G K S T Y
tr:Q3TZI5:Q3TZI5_MOUSE	V Q V A F I N P N M G G K S T Y
sp:P43247:MSH2_MOUSE	V Q V A F I N P N M G G K S T Y
tr:Q0IGW6:Q0IGW6_DROME	L Q V N F I N P N M G G K S T Y
sp:P43248:MSH2_DROME	L Q V N F I N P N M G G K S T Y
tr:Q9TXR4:Q9TXR4_CAEEL	I N K P F I N A N M G G K S T Y
sp:P25847:MSH2_YEAST	L Q I S F I N P N M G G K S T Y

protein	whole protein ident	simil	domain-based ident	simil	contact-based ident	simil
PDB:2o8b:chainA:P43246	1.0	1.0	1.0	1.0	1.0	1.0
PDB:2o8c:chainA:P43246	1.0	1.0	1.0	1.0	1.0	1.0
PDB:2o8d:chainA:P43246	1.0	1.0	1.0	1.0	1.0	1.0
PDB:2o8e:chainA:P43246	1.0	1.0	1.0	1.0	1.0	1.0
PDB:2o8f:chainA:P43246	1.0	1.0	1.0	1.0	1.0	1.0
PDB:3thw:chainA:P43246	1.0	1.0	1.0	1.0	1.0	1.0
PDB:3thx:chainA:P43246	1.0	1.0	1.0	1.0	1.0	1.0
PDB:3thy:chainA:P43246	1.0	1.0	1.0	1.0	1.0	1.0
PDB:3thz:chainA:P43246	1.0	1.0	1.0	1.0	1.0	1.0
tr:B4DL39:B4DL39_HUMAN	0.73	0.73	1.0	1.0	1.0	1.0
tr:E9PHA6:E9PHA6_HUMAN	0.94	0.97	1.0	1.0	1.0	1.0
sp:P43246:MSH2_HUMAN	1.0	1.0	1.0	1.0	1.0	1.0
tr:B1WBQ7:B1WBQ7_RAT	0.93	0.98	0.97	0.99	0.94	0.98
tr:Q3TZI5:Q3TZI5_MOUSE	0.92	0.98	0.97	0.99	0.94	0.98
sp:P43247:MSH2_MOUSE	0.92	0.98	0.97	0.99	0.94	0.98
tr:Q0IGW6:Q0IGW6_DROME	0.4	0.76	0.55	0.85	0.81	0.9
sp:P43248:MSH2_DROME	0.4	0.76	0.55	0.85	0.81	0.9
tr:Q9TXR4:Q9TXR4_CAEEL	0.28	0.67	0.49	0.82	0.69	0.87
sp:P25847:MSH2_YEAST	0.39	0.75	0.63	0.84	0.88	0.92

spel1 (UniProt:P43248) annotation
Function: Involved in postreplication mismatch repair. Binds specifically to DNA containing mismatched nucleotides thus providing a target for the excision repair processes characteristic of postreplication mismatch repair (By similarity).
Subunit: Heterodimer of Msh2/Spel and Msh6.
Subcellular location: Nucleus

(Information from UniProt)

msh-2 (WBGene00003418) associated phenotypes
radiation induced reproductive cell death variant

(Information from WormBase)

Glutathione synthetase

Best gene implication in ageing for this target family came from gene P46413 annotated in UniProt release 2014_02. Annotation GO 7568 (aging) was Inferred from Expression Pattern

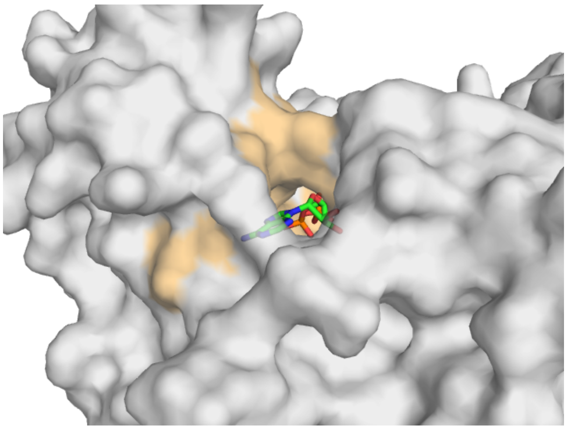

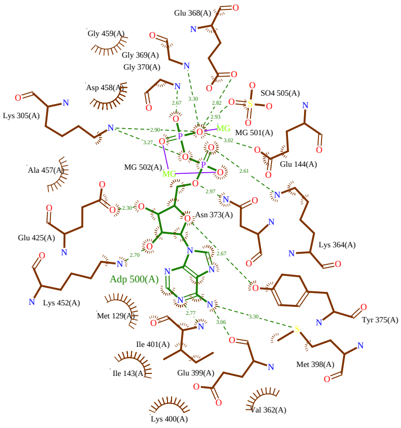

protein	amino acids contacts (binding site)
PDB:2hgs:chainA:P48637	M I E K V K E G G N Y M E K I E K A D G
tr:B7Z514:B7Z514_HUMAN	M I E K V K E G G N Y M E K I E K A D G
tr:B7Z1C5:B7Z1C5_HUMAN	M I E K V K E G G N Y M E K I E K A D G
sp:P48637:GSHB_HUMAN	M I E K V K E G G N Y M E K I E K A D G
sp:P46413:GSHB_RAT	M I E K V K E G G N Y M E K I E K A D G
tr:Q3UEE2:Q3UEE2_MOUSE	M I E K V K E G G N Y M E K I E K A D G
sp:P51855:GSHB_MOUSE	M I E K V K E G G N Y M E K I E K A D G
tr:Q541E2:Q541E2_MOUSE	M I E K V K E G G N Y M E K I E K A D G
tr:Q8IQZ1:Q8IQZ1_DROME	M V E K V K E G G N Y M D L I E K N E G
tr:A3FP35:A3FP35_DROME	M V E K V K E G G N Y M D L I E K N E G
tr:Q86B44:Q86B44_DROME	M V E K V K E G G N Y M D L I E K N E G
tr:Q21549:Q21549_CAEEL	M I E K V K E G G L T M E K L E K A M G
tr:E9P9G1:E9P9G1_YEASX	L V E K V K E G G N Y M E L I E K N E G
tr:E9P9L9:E9P9L9_YEASX	L V E K V K E G G N Y M E L I E K N E G
sp:Q08220:GSHB_YEAST	L V E K V K E G G N Y M E L I E K N E G

	whole protein		domain-based		contact-based	
protein	ident	simil	ident	simil	ident	simil
PDB:2hgs:chainA:P48637	1.0	1.0	1.0	1.0	1.0	1.0
tr:B7Z514:B7Z514_HUMAN	0.73	0.73	0.96	0.96	1.0	1.0
tr:B7Z1C5:B7Z1C5_HUMAN	0.85	0.85	1.0	1.0	1.0	1.0
sp:P48637:GSHB_HUMAN	1.0	1.0	1.0	1.0	1.0	1.0
sp:P46413:GSHB_RAT	0.88	0.97	0.93	0.98	1.0	1.0
tr:Q3UEE2:Q3UEE2_MOUSE	0.77	0.83	0.92	0.98	1.0	1.0
sp:P51855:GSHB_MOUSE	0.89	0.97	0.92	0.98	1.0	1.0
tr:Q541E2:Q541E2_MOUSE	0.89	0.97	0.92	0.98	1.0	1.0
tr:Q8IQZ1:Q8IQZ1_DROME	0.4	0.76	0.47	0.81	0.75	0.84
tr:A3FP35:A3FP35_DROME	0.4	0.77	0.46	0.8	0.75	0.84
tr:Q86B44:Q86B44_DROME	0.35	0.67	0.36	0.62	0.75	0.84
tr:Q21549:Q21549_CAEEL	0.38	0.75	0.44	0.79	0.8	0.81
tr:E9P9G1:E9P9G1_YEASX	0.34	0.72	0.41	0.78	0.75	0.86
tr:E9P9L9:E9P9L9_YEASX	0.34	0.72	0.41	0.78	0.75	0.86
sp:Q08220:GSHB_YEAST	0.34	0.72	0.41	0.78	0.75	0.86

GS (UniProt:Q8IQZ1) annotation

Cofactor: Mg(2+) Evidence=(PIRNR:PIRNR001558); Note=Binds 1 Mg(2+) ion per subunit. (PIRNR:PIRNR001558);
Pathway: Sulfur metabolism; glutathione biosynthesis; glutathione from L-cysteine and L-glutamate: step 2/2. (PIRNR:PIRNR001558).

(Information from UniProt)

UniProt:A3FP35 annotation

Cofactor: Mg(2+) Evidence=(PIRNR:PIRNR001558, PIRSR:PIRSR001558-2); Note=Binds 1 Mg(2+) ion per subunit. (PIRNR:PIRNR001558, PIRSR:PIRSR001558-2);
Pathway: Sulfur metabolism; glutathione biosynthesis; glutathione from L-cysteine and L-glutamate: step 2/2. (PIRNR:PIRNR001558).

(Information from UniProt)

gss-1 (WBGene00010941) associated phenotypes

lethal, sterile

(Information from WormBase)

gss-1 (UniProt:Q21549) annotation

Cofactor: Mg(2+) Evidence=(PIRNR:PIRNR001558, PIRSR:PIRSR001558-2); Note=Binds 1 Mg(2+) ion per subunit. (PIRNR:PIRNR001558, PIRSR:PIRSR001558-2);
Pathway: Sulfur metabolism; glutathione biosynthesis; glutathione from L-cysteine and L-glutamate: step 2/2. (PIRNR:PIRNR001558).

(Information from UniProt)

Glycogen synthase kinase-3 beta

Best gene implication in ageing for this target family came from gene P49841 via mapping the annotation from Ensembl ENSG00000082701 via mapping the annotation from EntrezGene 2932 via mapping the annotation from GenAgeHuman 0097 annotated in GenAge release 17.

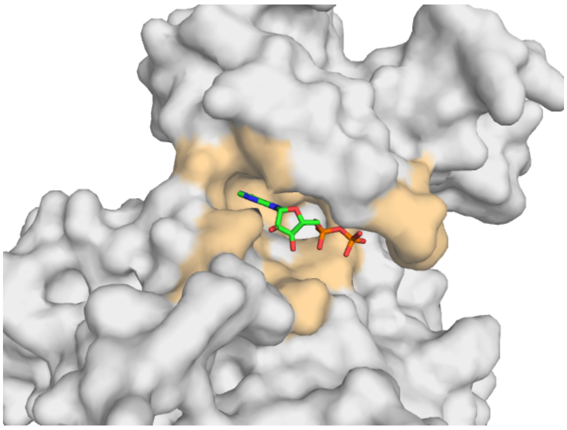

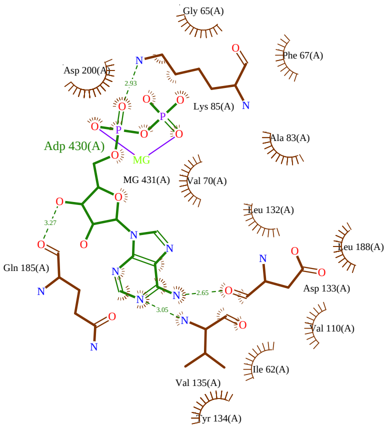

protein	amino acids contacts (binding site)
PDB:1j1c:chainA:P49841	I G S F V A K V L D Y V T R Q N L D
PDB:4nm0:chainA:P49841	I G S F V A K V L D Y V T R Q N L D
PDB:4nm3:chainA:P49841	I G S F V A K V L D Y V T R Q N L D
PDB:4nm5:chainA:P49841	I G S F V A K V L D Y V T R Q N L D
PDB:4nm7:chainA:P49841	I G S F V A K V L D Y V T R Q N L D
tr:Q6FI27:Q6FI27_HUMAN	I G S F V A K V L D Y V T R Q N L D
sp:P49841:GSK3B_HUMAN	I G S F V A K V L D Y V T R Q N L D
sp:P18266:GSK3B_RAT	I G S F V A K V L D Y V T R Q N L D
tr:Q5KU03:Q5KU03_MOUSE	I G S F V A K V L D Y V T R Q N L D
sp:Q9WV60:GSK3B_MOUSE	I G S F V A K V L D Y V T R Q N L D
tr:E9QAQ5:E9QAQ5_MOUSE	I G S F V A K V L D Y V T R Q N L D
tr:B6IDN4:B6IDN4_DROME	I G S F V A K V L E Y I T K Q N L D
sp:P18431:SGG_DROME	I G S F V A K V L E Y I T K Q N L D
tr:A4V3W2:A4V3W2_DROME	I G S F V A K V L E Y I T K Q N L D
tr:A4V3W1:A4V3W1_DROME	I G S F V A K V L E Y I T K Q N L D
tr:Q0KHW6:Q0KHW6_DROME	I G S F V A K V L E Y I T K Q N L D
tr:E6PBX9:E6PBX9_DROME	I G S F V A K V L E Y I T K Q N L D
tr:H5V852:H5V852_DROME	I G S F V A K V L E Y I T K Q N L D
sp:Q9U2Q9:GSK3_CAEEL	I G S F V A K V L E Y V T R Q N L D
sp:P50873:MRK1_YEAST	V G S F V A K V L D Y M S Q Q N L D

protein	whole protein ident	simil	domain-based ident	simil	contact-based ident	simil
PDB:1j1c:chainA:P49841	1.0	1.0	1.0	1.0	1.0	1.0
PDB:4nm0:chainA:P49841	1.0	1.0	1.0	1.0	1.0	1.0
PDB:4nm3:chainA:P49841	1.0	1.0	1.0	1.0	1.0	1.0
PDB:4nm5:chainA:P49841	1.0	1.0	1.0	1.0	1.0	1.0
PDB:4nm7:chainA:P49841	1.0	1.0	1.0	1.0	1.0	1.0
tr:Q6FI27:Q6FI27_HUMAN	1.0	1.0	1.0	1.0	1.0	1.0
sp:P49841:GSK3B_HUMAN	1.0	1.0	1.0	1.0	1.0	1.0
sp:P18266:GSK3B_RAT	0.99	1.0	1.0	1.0	1.0	1.0
tr:Q5KU03:Q5KU03_MOUSE	0.99	1.0	1.0	1.0	1.0	1.0
sp:Q9WV60:GSK3B_MOUSE	0.99	1.0	1.0	1.0	1.0	1.0
tr:E9QAQ5:E9QAQ5_MOUSE	0.96	0.97	0.96	0.96	1.0	1.0
tr:B6IDN4:B6IDN4_DROME	0.57	0.71	0.85	0.96	0.83	0.95
sp:P18431:SGG_DROME	0.57	0.73	0.85	0.96	0.83	0.95
tr:A4V3W2:A4V3W2_DROME	0.57	0.73	0.85	0.96	0.83	0.95
tr:A4V3W1:A4V3W1_DROME	0.51	0.65	0.85	0.96	0.83	0.95
tr:Q0KHW6:Q0KHW6_DROME	0.49	0.63	0.85	0.96	0.83	0.95
tr:E6PBX9:E6PBX9_DROME	0.19	0.26	0.52	0.67	0.83	0.95
tr:H5V852:H5V852_DROME	0.27	0.35	0.85	0.96	0.83	0.95
sp:Q9U2Q9:GSK3_CAEEL	0.61	0.78	0.81	0.95	0.94	0.98
sp:P50873:MRK1_YEAST	0.31	0.57	0.53	0.84	0.78	0.92

sgg (FBgn0003371) associated phenotypes

cell non-autonomous, cell polarity defective, chemical sensitive, circadian rhythm defective, developmental rate defective, germline clone, heat sensitive, lethal - all die before end of P-stage, lethal - all die before end of pupal stage, maternal effect, meiotic cell cycle defective, mitotic cell cycle defective, neuroanatomy defective, neurophysiology defective, non-rescuable maternal effect, planar polarity defective, rescuable maternal effect, short lived, smell perception defective, somatic clone, some die during pharate adult stage, some die during pupal stage, some die during third instar larval stage

(Information from FlyBase)

sgg (UniProt:P18431) annotation

Function: Required for several developmental events such as syncytial blastoderm formation and embryonic segmentation. Is involved in transcriptional regulation. Required for arm phosphorylation. Wg signaling operates by inactivating the sgg repression of en autoactivation. Negatively controls the neuromuscular junction (NMJ) growth in presynaptic motoneurons. Plays a role in the regulation of microtubule dynamics and actin cytoskeleton during embryogenesis. Required for phosphorylation of sra in activated eggs. Essential for completion of meiosis, possibly by triggering calcineurin activation via sra phosphorylation. Phosphorylates microtubule-associated protein futsch in axons. (PubMed:15269269, PubMed:16570248, PubMed:16949836, PubMed:2113617, PubMed:2118107, PubMed:22421435, PubMed:7529201, PubMed:8467811).
Subunit: Interacts with cos. (PubMed:1335365,PubMed:15691767).
Subcellular location: Cytoplasm. Nucleus. Cytoplasm, cytoskeleton, microtubule organizing center, centrosome. Cytoplasm, cell cortex. Cell junction, synapse. Cell projection, axon. Note=In syncytial embryos, detected at the centrosomes throughout the cell cycle, and in the mitotic spindle and pseudocleavage furrows invaginating from the cell cortex during mitosis. Concentrated at the growing end of membranes during the cellularization process. After cellularization, localized to the centrosomes during mitosis and to the nucleus at the end of telophase. Enriched in the presynaptic side of the neuromuscular junction, with some signal detected also in axonal branches.
Tissue specificity: Expressed in ovaries and activated eggs (at protein level). Expression is over all the embryo at all stages, no local accumulation is observed. (PubMed:2118107, PubMed:22421435, PubMed:8467811).
Developmental stage: Isoform SGG46 is expressed at low levels in 12-24 hours embryos. Isoform Zygotic and isoform SGG39 are expressed in 12-24 hours embryos and present throughout the larval, pupal and adult stages (at protein level). Isoform Zygotic is expressed maternally and zygotically but reduced throughout later embryonic development. Expression persists throughout larval stages.

(PubMed:2118107, PubMed:8467811).
Disruption phenotype: Mutants display an overdeveloped neuromuscular junction (NMJ), with the number of boutons greatly increased. (PubMed:15269269).

(Information from UniProt)

gsk-3 (WBGene00001746) associated phenotypes

axon morphology variant, larval lethal, lethal, locomotion variant, sterile

(Information from WormBase)

gsk-3 (UniProt:Q9U2Q9) annotation

Function: Phosphorylates oma-1, a regulator of the oocyte-to- embryo transition, enabling its degradation. Phosphorylates skn-1, preventing it from accumulating in nuclei and thus inhibiting phase II gene expression in the oxidative stress defense. Involved in mesendoderm specification and mitotic spindle orientation in EMS blastomeres. Thought to be a branch point in these processes as proteins downstream are not required. Negatively regulates Wnt signaling in vulval precursor cells and acts as a Wnt-independent repressor of med-1 and med-2 in the C lineage inhibiting mesoderm development. Required for normal lifespan and LiCl-induced lifespan extension. (PubMed:10444600, PubMed:11463373, PubMed:15572126, PubMed:16251270, PubMed:16289132, PubMed:16343905, PubMed:16930586, PubMed:17959600).
Subunit: Monomer (By similarity). Interacts with axl-1. (UniProtKB:Q9WV60, PubMed:17601533).
Disruption phenotype: Worms exhibit defects in endoderm specification and mitotic spindle alignment. Mutants show reduced degradation of oma-1 and have shortened lifespan. (PubMed:10444600).

(Information from UniProt)

DNA mismatch repair protein Msh6

Best gene implication in ageing for this target family came from gene Q9CRH0 annotated in UniProt release 2014_02. Annotation GO 8340 (determination of adult lifespan) was Inferred from Mutant Phenotype

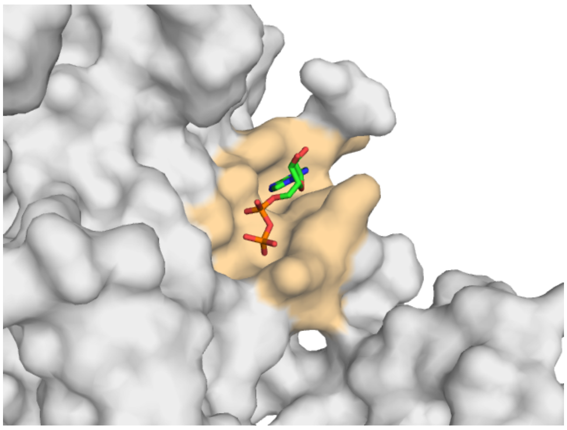

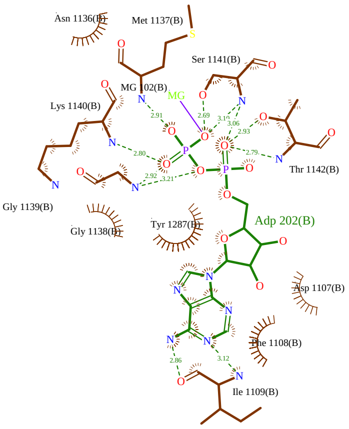

protein	amino acids contacts (binding site)
PDB:2o8b:chainB:P52701	D F I P N M G G K S T Y
PDB:2o8c:chainB:P52701	D F I P N M G G K S T Y
PDB:2o8d:chainB:P52701	D F I P N M G G K S T Y
sp:P52701:MSH6_HUMAN	D F I P N M G G K S T Y
tr:D4A0U9:D4A0U9_RAT	D F I P N M G G K S T Y
tr:Q9CRH0:Q9CRH0_MOUSE	D F I P N M G G K S T Y
sp:P54276:MSH6_MOUSE	D F I P N M G G K S T Y
sp:Q9VUM0:MSH6_DROME	T Y I P N M G G K S T Y
tr:Q9N3T8:Q9N3T8_CAEEL	S F I P N M G G K S T Y
sp:Q03834:MSH6_YEAST	D F I A N A A G K S T Y

	whole protein		domain-based		contact-based	
protein	ident	simil	ident	simil	ident	simil
PDB:2o8b:chainB:P52701	1.0	1.0	1.0	1.0	1.0	1.0
PDB:2o8c:chainB:P52701	1.0	1.0	1.0	1.0	1.0	1.0
PDB:2o8d:chainB:P52701	1.0	1.0	1.0	1.0	1.0	1.0
sp:P52701:MSH6_HUMAN	1.0	1.0	1.0	1.0	1.0	1.0
tr:D4A0U9:D4A0U9_RAT	0.86	0.95	0.94	0.98	1.0	1.0
tr:Q9CRH0:Q9CRH0_MOUSE	0.3	0.32	0.95	0.98	1.0	1.0
sp:P54276:MSH6_MOUSE	0.86	0.95	0.95	0.98	1.0	1.0
sp:Q9VUM0:MSH6_DROME	0.37	0.68	0.57	0.82	0.83	0.92
tr:Q9N3T8:Q9N3T8_CAEEL	0.3	0.63	0.51	0.79	0.92	0.95
sp:Q03834:MSH6_YEAST	0.25	0.58	0.43	0.75	0.75	0.87

Msh6 (UniProt:Q9VUM0) annotation

Function: Involved in post-replicative DNA-mismatch repair. (PubMed:17339220).
Subunit: Heterodimer of Msh2/Spel and Msh6.

(Information from UniProt)

Bloom syndrome protein

Best gene implication in ageing for this target family came from gene O18017 annotated in UniProt release 2014_02. Annotation GO 8340 (determination of adult lifespan) was Inferred from Mutant Phenotype

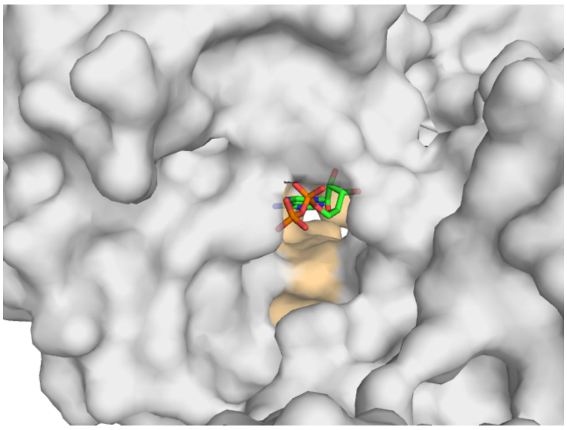

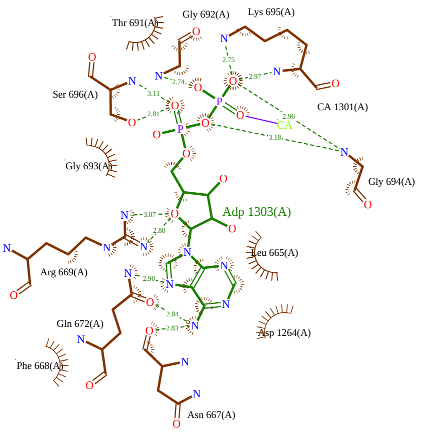

protein	amino acids contacts (binding site)
PDB:4cdg:chainA:P54132	L N F R Q P T G G G K S R N D
PDB:4cgz:chainA:P54132	L N F R Q P T G G G K S R N D
PDB:4o3m:chainA:P54132	L N F R Q P T G G G K S R N D
sp:P54132:BLM_HUMAN	L N F R Q P T G G G K S R N D
tr:D3ZQW1:D3ZQW1_RAT	L N F R Q P T G G G K S R N D
tr:E9PZ97:E9PZ97_MOUSE	L N F R Q P T G G G K S R N D
sp:O88700:BLM_MOUSE	L N F R Q P T G G G K S R N D
sp:Q9VGI8:BLM_DROME	L S F R Q P T G G G K S R N P
sp:O18017:BLM_CAEEL	F Q F R Q P T G A G K S R S D
sp:P35187:SGS1_YEAST	L G F R Q P T G G G K S R P G

	whole protein		domain-based		contact-based	
protein	ident	simil	ident	simil	ident	simil
PDB:4cdg:chainA:P54132	1.0	1.0	1.0	1.0	1.0	1.0
PDB:4cgz:chainA:P54132	1.0	1.0	1.0	1.0	1.0	1.0
PDB:4o3m:chainA:P54132	1.0	1.0	1.0	1.0	1.0	1.0
sp:P54132:BLM_HUMAN	1.0	1.0	1.0	1.0	1.0	1.0
tr:D3ZQW1:D3ZQW1_RAT	0.75	0.91	0.9	0.97	1.0	1.0
tr:E9PZ97:E9PZ97_MOUSE	0.76	0.91	0.92	0.97	1.0	1.0
sp:O88700:BLM_MOUSE	0.76	0.91	0.92	0.97	1.0	1.0
sp:Q9VGI8:BLM_DROME	0.27	0.62	0.46	0.77	0.87	0.9
sp:O18017:BLM_CAEEL	0.21	0.46	0.41	0.71	0.73	0.88
sp:P35187:SGS1_YEAST	0.21	0.55	0.32	0.65	0.8	0.82

Blm (FBgn0002906) associated phenotypes

DNA repair defective, chemical sensitive, dominant, increased cell death, maternal effect, mitotic cell cycle defective, partially, radiation sensitive

(Information from FlyBase)

Blm (UniProt:Q9VGI8) annotation

Function: Participates in DNA replication and repair. Exhibits a magnesium-dependent ATP-dependent DNA-helicase activity that unwinds single- and double-stranded DNA in a 3'-5' direction (By similarity).
Subcellular location: Nucleus

(Information from UniProt)

him-6 (WBGene00001865) associated phenotypes

XXX progeny, autosomal nondisjunction meiosis, embryonic lethal, germline mortal, high incidence male progeny, male fertility reduced, male sterile, nullo X gamete, reduced brood size

(Information from WormBase)

him-6 (UniProt:O18017) annotation

Function: Participates in DNA replication and repair. Exhibits a magnesium-dependent ATP-dependent DNA-helicase activity that unwinds single- and double-stranded DNA in a 3'-5' direction (By similarity).
Subcellular location: Nucleus

(Information from UniProt)

Transitional endoplasmic reticulum ATPase

Best gene implication in ageing for this target family came from gene P54811 via mapping the annotation from Ensembl WBGene00007352 via mapping the annotation from EntrezGene 174624 via mapping the annotation from GenAgeModels 1759 annotated in GenAge release 17.

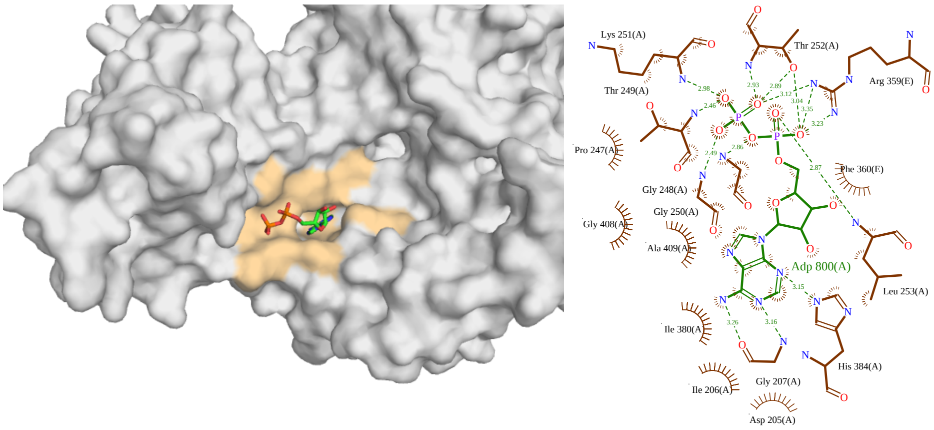

protein	amino acids contacts (binding site)														
PDB:4kod:chainA:P55072	D	I	G	P	G	T	G	K	T	L	R	I	H	G	A
tr:Q96IF9:Q96IF9_HUMAN	D	I	G	P	G	T	G	K	T	L	R	I	H	G	A
sp:P55072:TERA_HUMAN	D	I	G	P	G	T	G	K	T	L	R	I	H	G	A
sp:P46462:TERA_RAT	D	I	G	P	G	T	G	K	T	L	R	I	H	G	A
tr:Q8BNF8:Q8BNF8_MOUSE	D	I	G	P	G	T	G	K	T	L	R	I	H	G	A
sp:Q01853:TERA_MOUSE	D	I	G	P	G	T	G	K	T	L	R	I	H	G	A
sp:Q7KN62:TERA_DROME	D	I	G	P	G	T	G	K	T	L	R	V	H	G	A
tr:D0IQG4:D0IQG4_DROME	D	I	G	P	G	T	G	K	T	L	R	V	H	G	A
sp:P54811:TERA1_CAEEL	D	L	G	P	G	T	G	K	T	L	R	I	H	G	A
sp:P54812:TERA2_CAEEL	D	L	G	P	G	T	G	K	T	L	R	I	H	G	A
sp:P25694:CDC48_YEAST	D	I	G	P	G	T	G	K	T	L	R	V	H	G	A

	whole protein		domain-based		contact-based	
protein	ident	simil	ident	simil	ident	simil
PDB:4kod:chainA:P55072	1.0	1.0	1.0	1.0	1.0	1.0
tr:Q96IF9:Q96IF9_HUMAN	0.8	0.8	1.0	1.0	1.0	1.0
sp:P55072:TERA_HUMAN	1.0	1.0	1.0	1.0	1.0	1.0
sp:P46462:TERA_RAT	1.0	1.0	1.0	1.0	1.0	1.0
tr:Q8BNF8:Q8BNF8_MOUSE	0.9	0.9	1.0	1.0	1.0	1.0
sp:Q01853:TERA_MOUSE	1.0	1.0	1.0	1.0	1.0	1.0
sp:Q7KN62:TERA_DROME	0.83	0.94	0.88	0.97	0.93	0.98
tr:D0IQG4:D0IQG4_DROME	0.8	0.92	0.88	0.97	0.93	0.98
sp:P54811:TERA1_CAEEL	0.76	0.9	0.83	0.95	0.93	1.0
sp:P54812:TERA2_CAEEL	0.76	0.9	0.86	0.96	0.93	1.0
sp:P25694:CDC48_YEAST	0.66	0.87	0.83	0.96	0.93	0.98

TER94 (FBgn0261014) associated phenotypes

RU486 conditional, decreased cell death, flightless, increased cell death, learning defective, neuroanatomy defective, partially lethal - majority die, somatic clone, some die during pupal stage, stress response defective, temperature conditional

(Information from FlyBase)

TER94 (UniProt:Q7KN62) annotation

Function: Necessary for the fragmentation of Golgi stacks during mitosis and for their reassembly after mitosis. Involved in the formation of the transitional endoplasmic reticulum (tER). The transfer of membranes from the endoplasmic reticulum to the Golgi apparatus occurs via 50-70 nm transition vesicles which derive from part-rough, part-smooth transitional elements of the endoplasmic reticulum (tER). Vesicle budding from the tER is an ATP-dependent process. Involved in the ubiquitin-proteasome system. Important for oskar mRNA localization and/or anchoring during oogenesis. Involved in germ cell formation. (PubMed:14657277, PubMed:18590813).

Subunit: Homohexamer. Interacts with tud, vas, papi and AGO3.(PubMed:10564274, PubMed:18590813, PubMed:21447556).

Subcellular location: Cytoplasm. Nucleus. Note=Component of the meiotic nuage, also named P granule, a germ-cell-specific organelle required to repress transposon activity during meiosis.

Tissue specificity: Present in the mushroom bodies of the protocerebrum and in the glomeruli of the antennal lobe. Present in nurse cells, oocytes and sperm bundles (at protein level). (PubMed:10564274, PubMed:10656772, PubMed:9639875).
Developmental stage: Present in egg, pupa and imago but not larva (at protein level). (PubMed:9639875).

(Information from UniProt)

cdc-48.1 (WBGene00007352) associated phenotypes
tunicamycin hypersensitive, unfolded protein response variant

(Information from WormBase)

cdc-48.2 (WBGene00008053) associated phenotypes
reduced brood size

(Information from WormBase)

5'-AMP-activated protein kinase subunit gamma-1

Best gene implication in ageing for this target family came from gene P12904 annotated in UniProt release 2014_02. Annotation GO subterm of 7568 (aging) was Inferred from Mutant Phenotype

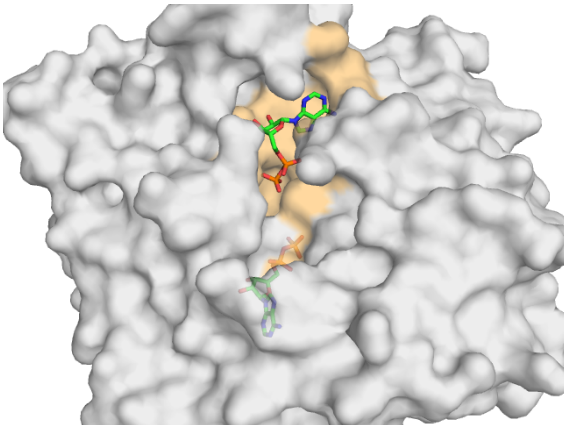

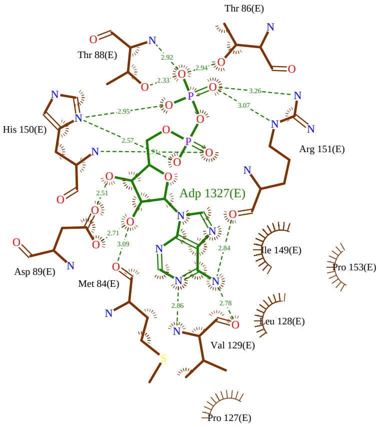

protein	amino acids contacts (binding site)
PDB:2y8l:chainE:P80385	R M T I T D P L V I H R P K I S F D R G V L V H R
PDB:2y8q:chainE:P80385	R M T I T D P L V I H R P K I S F D R G V L V H R
tr:F8VYY9:F8VYY9_HUMAN	R M T I T D P L V I H R P K I S F D R G V L V H R
sp:P54619:AAKG1_HUMAN	R M T I T D P L V I H R P K I S F D R G V L V H R
sp:P80385:AAKG1_RAT	R M T I T D P L V I H R P K I S F D R G V L V H R
sp:O54950:AAKG1_MOUSE	R M T I T D P L V I H R P K I S F D R G V L V H R
tr:Q3TWR3:Q3TWR3_MOUSE	R M T I T D P L V I H R P K I S F D R G V L V H R
tr:Q7KNS9:Q7KNS9_DROME	R M T I T D P L V I H R P K I A F D R G V Q V H R
tr:Q8IGX0:Q8IGX0_DROME	R M T I T D P L V I H R P K I A F D R G V Q V H R
tr:Q0KI34:Q0KI34_DROME	R M T I T D P L V I H R P K I A F D R G V Q V H R
tr:A1A703:A1A703_DROME	R M T I T D P L V I H R P K I A F D R G V Q V H R
tr:A1A6Y3:A1A6Y3_DROME	R M T I T D P L V I H R P K I A F D R G V Q V H R
tr:O96613:O96613_DROME	R M T I T D P L V I H R P K I A F D R G V Q V H R
tr:Q8I0K6:Q8I0K6_DROME	R M T I T D P L V I H R P K I A F D R G V Q V H R
tr:Q8I0A1:Q8I0A1_DROME	R M T I T D P L V I H R P K I A F D R G V Q V H R
tr:Q8SXT8:Q8SXT8_DROME	R M T I T D P L V I H R P K I A F D R G V Q V H R
tr:Q8I0B2:Q8I0B2_DROME	R M T I T D P L V I H R P K I A F D R G V Q V H R
tr:Q7JQX9:Q7JQX9_DROME	R M T I T D P L V I H R P K I A F D R G V Q V H R
tr:Q9VDD2:Q9VDD2_DROME	R M T I T D P L V I H R P K I A F D R G V Q V H R
tr:Q9BHL6:Q9BHL6_CAEEL	R M T I T D P F V V H R P K I A F D R G V Q V H R
sp:P12904:AAKG_YEAST	V L T T T D D T A S G R P Y V E Y D R G V Y V H R
tr:F4ZZV2:F4ZZV2_YEASX	V L T T T D D T A S G R P Y V E Y D R G V Y V H R

protein	whole protein ident	whole protein simil	domain-based ident	domain-based simil	contact-based ident	contact-based simil
PDB:2y8l:chainE:P80385	1.0	1.0	1.0	1.0	1.0	1.0
PDB:2y8q:chainE:P80385	1.0	1.0	1.0	1.0	1.0	1.0
tr:F8VYY9:F8VYY9_HUMAN	0.83	0.84	0.86	0.87	1.0	1.0
sp:P54619:AAKG1_HUMAN	0.96	0.99	0.98	1.0	1.0	1.0
sp:P80385:AAKG1_RAT	1.0	1.0	1.0	1.0	1.0	1.0
sp:O54950:AAKG1_MOUSE	0.98	0.99	0.98	0.99	1.0	1.0
tr:Q3TWR3:Q3TWR3_MOUSE	0.98	0.99	0.98	0.99	1.0	1.0
tr:Q7KNS9:Q7KNS9_DROME	0.39	0.54	0.64	0.87	0.92	0.92
tr:Q8IGX0:Q8IGX0_DROME	0.37	0.53	0.63	0.88	0.92	0.92
tr:Q0KI34:Q0KI34_DROME	0.37	0.53	0.63	0.88	0.92	0.92
tr:A1A703:A1A703_DROME	0.32	0.46	0.64	0.88	0.92	0.92
tr:A1A6Y3:A1A6Y3_DROME	0.32	0.46	0.63	0.88	0.92	0.92
tr:O96613:O96613_DROME	0.31	0.44	0.64	0.88	0.92	0.92
tr:Q8I0K6:Q8I0K6_DROME	0.28	0.4	0.64	0.88	0.92	0.92
tr:Q8I0A1:Q8I0A1_DROME	0.25	0.35	0.64	0.88	0.92	0.92
tr:Q8SXT8:Q8SXT8_DROME	0.22	0.31	0.64	0.88	0.92	0.92
tr:Q8I0B2:Q8I0B2_DROME	0.22	0.31	0.64	0.88	0.92	0.92
tr:Q7JQX9:Q7JQX9_DROME	0.21	0.3	0.64	0.88	0.92	0.92
tr:Q9VDD2:Q9VDD2_DROME	0.14	0.2	0.64	0.88	0.92	0.92
tr:Q9BHL6:Q9BHL6_CAEEL	0.3	0.47	0.54	0.83	0.84	0.9
sp:P12904:AAKG_YEAST	0.3	0.7	0.33	0.75	0.48	0.64
tr:F4ZZV2:F4ZZV2_YEASX	0.3	0.7	0.33	0.75	0.48	0.64

SNF4Agamma (FBgn0264357) associated phenotypes

feeding behavior defective, hyperactive, neuroanatomy defective, nutrition conditional, phototaxis defective, short lived, starvation stress response defective

(Information from FlyBase)

Transitional endoplasmic reticulum ATPase

Best gene implication in ageing for this target family came from gene P54811 via mapping the annotation from Ensembl WBGene00007352 via mapping the annotation from EntrezGene 174624 via mapping the annotation from GenAgeModels 1759 annotated in GenAge release 17.

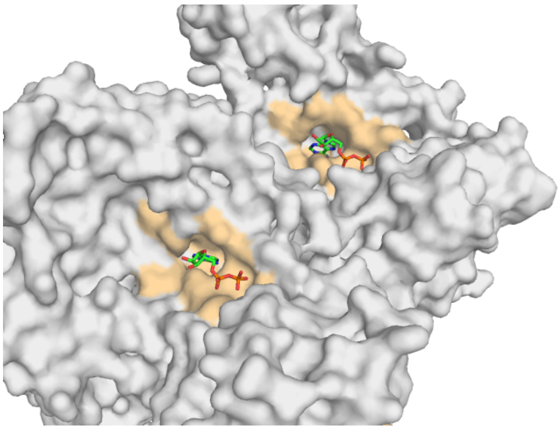

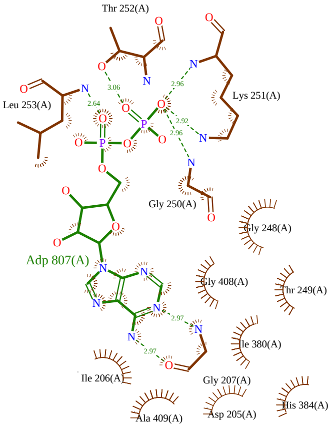

protein	amino acids contacts (binding site)
PDB:1e32:chainA:Q01853	D V G C P G T G K T L D R I H G A - - - - - - - - - - - - - - - - -
PDB:1r7r:chainA:Q01853	D V G C P G T G K T L D R I H G A D I G L P P G C G K T L I N G A T
PDB:1s3s:chainA:Q01853	D V G C P G T G K T L D R I H G A - - - - - - - - - - - - - - - - -
PDB:3cf0:chainA:Q01853	- - - - - - - - - - - - - - - - - D I G L P P G C G K T L I N G A T
PDB:3cf1:chainA:Q01853	D I G C P G T G K T L D R I H G A D I G L P P G C G K T L I N G A T
PDB:3cf2:chainA:Q01853	D I G C P G T G K T L D R I H G A D I G L P P G C G K T L I N - - T
PDB:3cf3:chainA:Q01853	D I G C P G T G K T L D R I H G A D I G L P P G C G K T L I N G A T
tr:Q96IF9:Q96IF9_HUMAN	D I G C P G T G K T L D R I H G A D I G L P P G C G K T L I N G A T
sp:P55072:TERA_HUMAN	D I G C P G T G K T L D R I H G A D I G L P P G C G K T L I N G A T
sp:P46462:TERA_RAT	D I G C P G T G K T L D R I H G A D I G L P P G C G K T L I N G A T
tr:Q8BNF8:Q8BNF8_MOUSE	D I G C P G T G K T L D R I H G A D I G L P P G C G K T L I N G A T
sp:Q01853:TERA_MOUSE	D I G C P G T G K T L D R I H G A D I G L P P G C G K T L I N G A T
sp:Q7KN62:TERA_DROME	D I G C P G T G K T L D R V H G A D I G L P P G C G K T L I N G A T
tr:D0IQG4:D0IQG4_DROME	D I G C P G T G K T L D R V H G A D I G L P P G C G K T L I N G A T
sp:P54811:TERA1_CAEEL	D L G V P G T G K T L D R I H G A D I G L P P G C G K T L I S G A T
sp:P54812:TERA2_CAEEL	D L G V P G T G K T L D R I H G A D I G L P P G C G K T L I S G A T
sp:P25694:CDC48_YEAST	D I G C P G T G K T L D R V H G A D V G L P P G T G K T L I Q G A L

protein	whole protein ident	whole protein simil	domain-based ident	domain-based simil	contact-based ident	contact-based simil
PDB:1e32:chainA:Q01853	1.0	1.0	1.0	1.0	0.5	0.0
PDB:1r7r:chainA:Q01853	1.0	1.0	1.0	1.0	1.0	1.0
PDB:1s3s:chainA:Q01853	1.0	1.0	1.0	1.0	0.5	0.0
PDB:3cf0:chainA:Q01853	1.0	1.0	1.0	1.0	0.5	0.0
PDB:3cf1:chainA:Q01853	1.0	1.0	1.0	1.0	0.97	0.99
PDB:3cf2:chainA:Q01853	0.99	1.0	1.0	1.0	0.91	0.87
PDB:3cf3:chainA:Q01853	1.0	1.0	1.0	1.0	0.97	0.99
tr:Q96IF9:Q96IF9_HUMAN	0.8	0.8	1.0	1.0	0.97	0.99
sp:P55072:TERA_HUMAN	1.0	1.0	1.0	1.0	0.97	0.99
sp:P46462:TERA_RAT	1.0	1.0	1.0	1.0	0.97	0.99
tr:Q8BNF8:Q8BNF8_MOUSE	0.9	0.9	1.0	1.0	0.97	0.99
sp:Q01853:TERA_MOUSE	1.0	1.0	1.0	1.0	0.97	0.99
sp:Q7KN62:TERA_DROME	0.83	0.94	0.88	0.97	0.94	0.98
tr:D0IQG4:D0IQG4_DROME	0.8	0.92	0.88	0.97	0.94	0.98
sp:P54811:TERA1_CAEEL	0.77	0.91	0.83	0.94	0.91	0.93
sp:P54812:TERA2_CAEEL	0.77	0.91	0.84	0.95	0.91	0.93
sp:P25694:CDC48_YEAST	0.66	0.87	0.74	0.91	0.82	0.9

TER94 (FBgn0261014) associated phenotypes

RU486 conditional, decreased cell death, flightless, increased cell death, learning defective, neuroanatomy defective, partially lethal - majority die, somatic clone, some die during pupal stage, stress response defective, temperature conditional

(Information from FlyBase)

TER94 (UniProt:Q7KN62) annotation

Function: Necessary for the fragmentation of Golgi stacks during mitosis and for their reassembly after mitosis. Involved in the formation of the transitional endoplasmic reticulum (tER). The transfer of membranes from the endoplasmic reticulum to the Golgi apparatus occurs via 50-70 nm transition vesicles which derive from part-rough, part-smooth transitional elements of the endoplasmic reticulum (tER). Vesicle budding from the tER is an ATP-dependent process. Involved in the ubiquitin-proteasome system. Important for oskar mRNA localization and/or anchoring during oogenesis. Involved in germ cell formation. (PubMed:14657277, PubMed:18590813).
Subunit: Homohexamer. Interacts with tud, vas, papi and AGO3.(PubMed:10564274, PubMed:18590813, PubMed:21447556).
Subcellular location: Cytoplasm. Nucleus. Note=Component of the meiotic nuage, also named P granule, a germ-cell-specific organelle required to repress transposon activity during meiosis.
Tissue specificity: Present in the mushroom bodies of the protocerebrum and in the glomeruli of the antennal lobe. Present in nurse cells, oocytes and sperm bundles (at protein level). (PubMed:10564274, PubMed:10656772, PubMed:9639875).
Developmental stage: Present in egg, pupa and imago but not larva (at protein level). (PubMed:9639875).

(Information from UniProt)

cdc-48.1 (WBGene00007352) associated phenotypes
tunicamycin hypersensitive, unfolded protein response variant

(Information from WormBase)

cdc-48.2 (WBGene00008053) associated phenotypes
reduced brood size

(Information from WormBase)

Dual specificity mitogen-activated protein kinase kinase 1

Best gene implication in ageing for this target family came from gene Q02750 annotated in UniProt release 2014_02. Annotation GO subterm of 7568 (aging) was Inferred from Mutant Phenotype

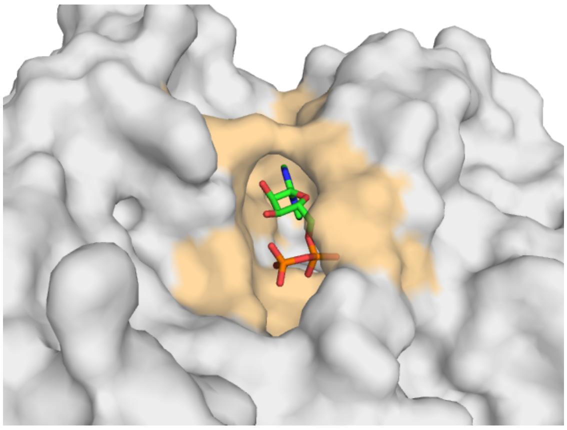

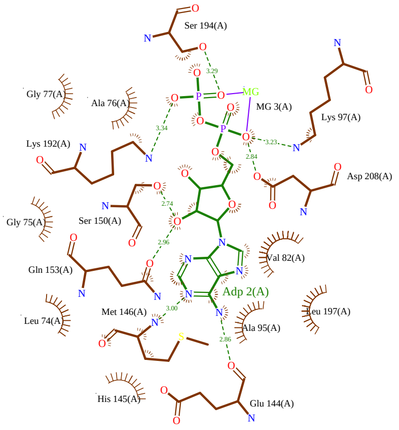

protein	amino acids contacts (binding site)
PDB:3eqg:chainA:Q02750	L G A G G V A K V M E H M S D Q K S N L D
PDB:3eqh:chainA:Q02750	L G A G G V A K V M E H M S D Q K S N L D
PDB:3eqi:chainA:Q02750	L G A G G V A K V M E H M S D Q K S N L D
PDB:3mbl:chainA:Q02750	L G A G G V A K V M E H M S D Q K S N L D
PDB:4ark:chainA:Q02750	L G A G G V A K V M E H M S D Q K S N L D
sp:Q02750:MP2K1_HUMAN	L G A G G V A K V M E H M S D Q K S N L D
tr:A4QPA9:A4QPA9_HUMAN	L G A G G V A K V M E H M S D Q K S N L D
sp:Q01986:MP2K1_RAT	L G A G G V A K V M E H M S D Q K S N L D
tr:Q3TMJ8:Q3TMJ8_MOUSE	L G A G G V A K V M E H M S D Q K S N L D
sp:P31938:MP2K1_MOUSE	L G A G G V A K V M E H M S D Q K S N L D
sp:Q24324:DSOR1_DROME	L G S G G V A K V M E Y M S D L K S N L D
sp:Q10664:MEK2_CAEEL	L G H G G V A K V M E Y M S D I K S N L D
sp:P08018:PBS2_YEAST	L G H G G V A K V M E Y M S D K K T N L D

	whole protein		domain-based		contact-based	
protein	ident	simil	ident	simil	ident	simil
PDB:3eqg:chainA:Q02750	1.0	1.0	1.0	1.0	1.0	1.0
PDB:3eqh:chainA:Q02750	1.0	1.0	1.0	1.0	1.0	1.0
PDB:3eqi:chainA:Q02750	1.0	1.0	1.0	1.0	1.0	1.0
PDB:3mbl:chainA:Q02750	1.0	1.0	1.0	1.0	1.0	1.0
PDB:4ark:chainA:Q02750	1.0	1.0	1.0	1.0	1.0	1.0
sp:Q02750:MP2K1_HUMAN	1.0	1.0	1.0	1.0	1.0	1.0
tr:A4QPA9:A4QPA9_HUMAN	1.0	1.0	1.0	1.0	1.0	1.0
sp:Q01986:MP2K1_RAT	0.99	1.0	1.0	1.0	1.0	1.0
tr:Q3TMJ8:Q3TMJ8_MOUSE	0.99	1.0	1.0	1.0	1.0	1.0
sp:P31938:MP2K1_MOUSE	0.99	1.0	1.0	1.0	1.0	1.0
sp:Q24324:DSOR1_DROME	0.6	0.82	0.75	0.93	0.86	0.86
sp:Q10664:MEK2_CAEEL	0.47	0.74	0.62	0.88	0.86	0.87
sp:P08018:PBS2_YEAST	0.2	0.36	0.45	0.74	0.81	0.87

Dsor1 (FBgn0010269) associated phenotypes

dominant, lethal - all die before end of pupal stage, neuroanatomy defective, polyphasic, some die during immature adult stage, some die during pupal stage

(Information from FlyBase)

Dsor1 (UniProt:Q24324) annotation

Function: Required downstream of Raf in the sevenless (sev), torso (tor), and Drosophila EGF receptor homolog (DER) signal transduction pathways. Involved in both positive regulation (at the posterior terminus) and negative regulation (at the anterior domain) of tll, as in other terminal class gene products, maybe via the ERK-A kinase. (PubMed:8381718).
Developmental stage: Expressed both maternally and zygotically. (PubMed:8381718).
Ptm: Phosphorylation on Ser/Thr by MAP kinase kinase kinases regulates positively the kinase activity.

(Information from UniProt)

mek-2 (WBGene00003186) associated phenotypes

AWA odorant chemotaxis defective, AWC odorant chemotaxis defective, bacterially unswollen, constipated, germ cell arrest, germ nuclei rachis, lethal, metabolic pathway variant, no oocytes, pachytene region organization variant, pathogen susceptibility increased, rod like larval lethal, sterile, vulval cell lineage variant, vulvaless

(Information from WormBase)

mek-2 (UniProt:Q10664) annotation

Function: Functions in the let-60 Ras signaling pathway; acts downstream of lin-45 raf kinase, but before the sur-1/mpK-1 gene product in controlling vulval cell differentiation.
Enzyme regulation: Activated by tyrosine and threoninephosphorylation catalyzed by MAP kinase kinase kinases.

(Information from UniProt)

Focal adhesion kinase 1

Best gene implication in ageing for this target family came from gene Q05397 via mapping the annotation from Ensembl ENSG00000169398 via mapping the annotation from EntrezGene 5747 via mapping the annotation from GenAgeHuman 0166 annotated in GenAge release 17.

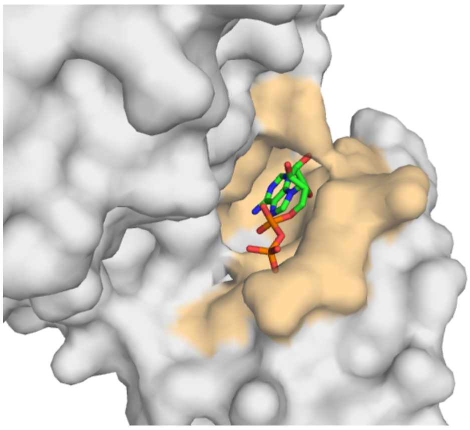

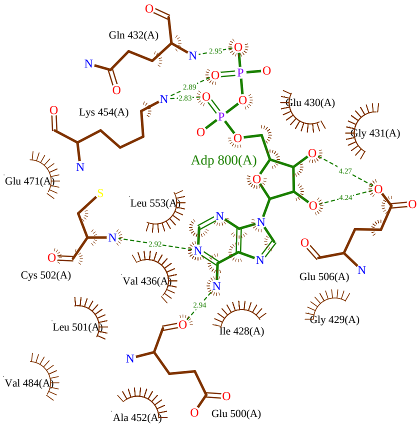

protein	amino acids contacts (binding site)														
PDB:1mp8:chainA:Q05397	I	G	E	G	Q	V	A	K	E	V	E	L	C	E	L
tr:E9PEI4:E9PEI4_HUMAN	I	G	E	G	Q	V	A	K	E	V	E	L	C	E	L
tr:H0YB16:H0YB16_HUMAN	I	G	E	G	Q	V	A	K	E	V	E	L	C	E	L
tr:Q8N9D7:Q8N9D7_HUMAN	I	G	E	G	Q	V	A	K	E	V	E	L	C	E	L
tr:B4DH13:B4DH13_HUMAN	I	G	E	G	Q	V	A	K	E	V	E	L	C	E	L
tr:H0YBZ1:H0YBZ1_HUMAN	I	G	E	G	Q	V	A	K	E	V	E	L	C	E	L
tr:Q8IYN9:Q8IYN9_HUMAN	I	G	E	G	Q	V	A	K	E	V	E	L	C	E	L
sp:Q05397:FAK1_HUMAN	I	G	E	G	Q	V	A	K	E	V	E	L	C	E	L
tr:J3QT16:J3QT16_HUMAN	I	G	E	G	Q	V	A	K	E	V	E	L	C	E	L
tr:E7ESA6:E7ESA6_HUMAN	I	G	E	G	Q	V	A	K	E	V	E	L	C	E	L
tr:R9PXT6:R9PXT6_RAT	I	G	E	G	Q	V	A	K	E	V	E	L	C	E	L
sp:P34152:FAK1_MOUSE	I	G	E	G	Q	V	A	K	E	V	E	L	C	E	L
tr:Q2TGI0:Q2TGI0_DROME	I	G	V	G	Q	V	A	K	E	I	E	L	A	E	L
tr:Q9U5Y2:Q9U5Y2_DROME	I	G	V	G	Q	V	A	K	E	I	E	L	A	E	L
tr:E1JGM8:E1JGM8_DROME	I	G	V	G	Q	V	A	K	E	I	E	L	A	E	L
tr:B8A418:B8A418_DROME	I	G	V	G	Q	V	A	K	E	I	E	L	A	E	L
tr:Q5BIG9:Q5BIG9_DROME	I	G	V	G	Q	V	A	K	E	I	E	L	A	E	L
tr:Q0E917:Q0E917_DROME	I	G	V	G	Q	V	A	K	E	I	E	L	A	E	L
tr:Q9U531:Q9U531_DROME	I	G	V	G	Q	V	A	K	E	I	E	L	A	E	L
tr:Q9U472:Q9U472_DROME	I	G	V	G	Q	V	A	K	E	I	E	L	A	E	L
tr:B7YZL9:B7YZL9_DROME	I	G	V	G	Q	V	A	K	E	I	E	L	A	E	L
tr:Q8T879:Q8T879_CAEEL	I	G	G	G	Q	V	A	K	E	I	E	L	A	E	L
tr:Q95YD4:Q95YD4_CAEEL	I	G	G	G	Q	V	A	K	E	I	E	L	A	E	L

protein	whole protein ident	simil	domain-based ident	simil	contact-based ident	simil
PDB:1mp8:chainA:Q05397	1.0	1.0	1.0	1.0	1.0	1.0
tr:E9PEI4:E9PEI4_HUMAN	0.63	0.64	1.0	1.0	1.0	1.0
tr:H0YB16:H0YB16_HUMAN	0.68	0.68	0.97	0.97	1.0	1.0
tr:Q8N9D7:Q8N9D7_HUMAN	0.67	0.67	0.97	0.97	1.0	1.0
tr:B4DH13:B4DH13_HUMAN	0.65	0.65	0.98	0.98	1.0	1.0
tr:H0YBZ1:H0YBZ1_HUMAN	0.66	0.66	0.98	0.98	1.0	1.0
tr:Q8IYN9:Q8IYN9_HUMAN	0.96	0.96	1.0	1.0	1.0	1.0
sp:Q05397:FAK1_HUMAN	1.0	1.0	1.0	1.0	1.0	1.0
tr:J3QT16:J3QT16_HUMAN	0.99	0.99	1.0	1.0	1.0	1.0
tr:E7ESA6:E7ESA6_HUMAN	0.96	0.96	1.0	1.0	1.0	1.0
tr:R9PXT6:R9PXT6_RAT	0.97	0.99	0.99	1.0	1.0	1.0
sp:P34152:FAK1_MOUSE	0.94	0.96	0.95	0.95	1.0	1.0
tr:Q2TGI0:Q2TGI0_DROME	0.16	0.23	0.56	0.79	0.8	0.77
tr:Q9U5Y2:Q9U5Y2_DROME	0.3	0.62	0.56	0.79	0.8	0.77
tr:E1JGM8:E1JGM8_DROME	0.3	0.62	0.56	0.79	0.8	0.77
tr:B8A418:B8A418_DROME	0.3	0.62	0.56	0.79	0.8	0.77
tr:Q5BIG9:Q5BIG9_DROME	0.3	0.62	0.56	0.79	0.8	0.77
tr:Q0E917:Q0E917_DROME	0.3	0.62	0.56	0.79	0.8	0.77
tr:Q9U531:Q9U531_DROME	0.3	0.62	0.56	0.79	0.8	0.77
tr:Q9U472:Q9U472_DROME	0.3	0.62	0.56	0.79	0.8	0.77
tr:B7YZL9:B7YZL9_DROME	0.25	0.52	0.56	0.79	0.8	0.77
tr:Q8T879:Q8T879_CAEEL	0.21	0.53	0.47	0.77	0.8	0.79
tr:Q95YD4:Q95YD4_CAEEL	0.21	0.55	0.47	0.77	0.8	0.79

Fak (FBgn0020440) associated phenotypes
neuroanatomy defective, neurophysiology defective

(Information from FlyBase)

ATP-dependent RNA helicase A

Best gene implication in ageing for this target family came from gene P24785 annotated in UniProt release 2014_02. Annotation GO 8340 (determination of adult lifespan) was Inferred from Mutant Phenotype

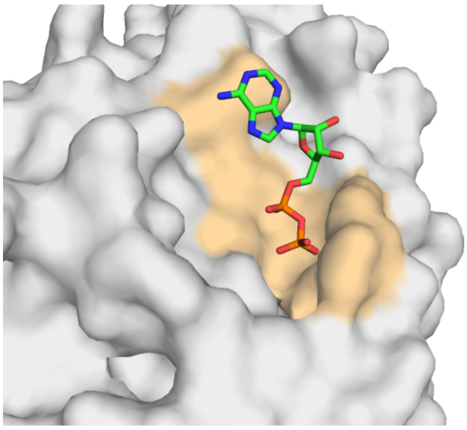

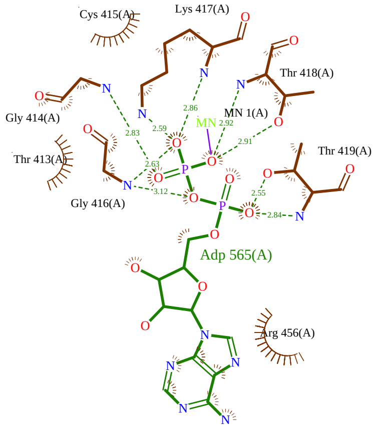

protein	amino acids contacts (binding site)
PDB:3llm:chainA:Q08211	T G C G K T T R
sp:Q08211:DHX9_HUMAN	T G C G K T T R
tr:D4A9D6:D4A9D6_RAT	T G C G K T T R
tr:Q3UR42:Q3UR42_MOUSE	T G C G K T T R
tr:E9QNN1:E9QNN1_MOUSE	T G C G K T T R
tr:I1V4Y3:I1V4Y3_DROME	T G C G K T T R
tr:B3STG5:B3STG5_DROME	T G C G K T T R
tr:A8ILA7:A8ILA7_DROME	T G C G K T T R
tr:B3STH6:B3STH6_DROME	T G C G K T T R
tr:B3STH2:B3STH2_DROME	T G C G K T T R
tr:B3STH3:B3STH3_DROME	T G C G K T T R
tr:B3STH0:B3STH0_DROME	T G C G K T T R
tr:A8IL99:A8IL99_DROME	T G C G K T T R
sp:P24785:MLE_DROME	T G C G K T T R
tr:B3STG3:B3STG3_DROME	T G C G K T T R
tr:B3STG2:B3STG2_DROME	T G C G K T T R
tr:B3STG7:B3STG7_DROME	T G C G K T T R
tr:B3STG6:B3STG6_DROME	T G C G K T T R
tr:B3STG4:B3STG4_DROME	T G C G K T T R
tr:B3STG9:B3STG9_DROME	T G C G K T T R
tr:A8ILB3:A8ILB3_DROME	T G C G K T T R
sp:Q22307:DHX9_CAEEL	T G C G K S T R

	whole protein		domain-based		contact-based	
protein	ident	simil	ident	simil	ident	simil
PDB:3llm:chainA:Q08211	1.0	1.0	1.0	1.0	1.0	1.0
sp:Q08211:DHX9_HUMAN	1.0	1.0	1.0	1.0	1.0	1.0
tr:D4A9D6:D4A9D6_RAT	0.86	0.9	0.97	0.99	1.0	1.0
tr:Q3UR42:Q3UR42_MOUSE	0.53	0.56	0.96	0.99	1.0	1.0
tr:E9QNN1:E9QNN1_MOUSE	0.85	0.9	0.96	0.99	1.0	1.0
tr:I1V4Y3:I1V4Y3_DROME	0.36	0.55	0.68	0.91	1.0	1.0
tr:B3STG5:B3STG5_DROME	0.47	0.76	0.68	0.91	1.0	1.0
tr:A8ILA7:A8ILA7_DROME	0.46	0.76	0.68	0.91	1.0	1.0
tr:B3STH6:B3STH6_DROME	0.47	0.76	0.68	0.91	1.0	1.0
tr:B3STH2:B3STH2_DROME	0.47	0.76	0.68	0.91	1.0	1.0
tr:B3STH3:B3STH3_DROME	0.47	0.76	0.68	0.91	1.0	1.0
tr:B3STH0:B3STH0_DROME	0.47	0.76	0.68	0.91	1.0	1.0
tr:A8IL99:A8IL99_DROME	0.46	0.76	0.68	0.91	1.0	1.0
sp:P24785:MLE_DROME	0.47	0.76	0.68	0.91	1.0	1.0
tr:B3STG3:B3STG3_DROME	0.47	0.76	0.68	0.91	1.0	1.0
tr:B3STG2:B3STG2_DROME	0.47	0.76	0.68	0.91	1.0	1.0
tr:B3STG7:B3STG7_DROME	0.47	0.76	0.68	0.91	1.0	1.0
tr:B3STG6:B3STG6_DROME	0.47	0.76	0.68	0.91	1.0	1.0
tr:B3STG4:B3STG4_DROME	0.47	0.76	0.68	0.91	1.0	1.0
tr:B3STG9:B3STG9_DROME	0.47	0.76	0.68	0.91	1.0	1.0
tr:A8ILB3:A8ILB3_DROME	0.47	0.76	0.68	0.91	1.0	1.0
sp:Q22307:DHX9_CAEEL	0.38	0.71	0.62	0.9	0.88	0.93

mle (FBgn0002774) associated phenotypes

chemical resistant, chemical sensitive, heat sensitive, locomotor behavior defective, male fertile, male limited, neuroanatomy defective, neurophysiology defective, paralytic, partially, partially lethal - majority die, partially lethal - majority live, poor, short lived, some die during pupal stage, some die during third instar larval stage, song defective

(Information from FlyBase)

mle (UniProt:P24785) annotation

Function: Required in males for dosage compensation of X chromosome linked genes. Mle, msl-1 and msl-3 are colocalized on X chromosome. Each of the msl proteins requires all the other msls for wild-type X-chromosome binding. Probably unwinds double- stranded DNA and RNA in a 3' to 5' direction. (PubMed:1653648).

Subcellular location: Nucleus (PubMed:1653648).Chromosome (PubMed:1653648). Note=Mle is associated with hundreds of discrete sites along the length of the X chromosome in males and not in females, and is associated with 30- 40 autosomal sites in both sexes.
Developmental stage: Expressed throughout development; highest in embryos and at equal levels in males and females. (PubMed:1653648).

(Information from UniProt)

rha-1 (UniProt:Q22307) annotation

Function: Unwinds double-stranded DNA and RNA in a 3' to 5' direction. Generates multiple secondary structures that influence RNA-binding proteins (By similarity). Transcriptional regulator that controls germline mitosis and development via histone modification. (, PubMed:15817227).
Subcellular location: Nucleus
Tissue specificity: Expressed in the pachytene region of the gonad and in the oocytes. (PubMed:15817227).

(Information from UniProt)

Tyrosine-protein kinase ITK/TSK

Best gene implication in ageing for this target family came from gene P08630 annotated in UniProt release 2014_02. Annotation GO 8340 (determination of adult lifespan) was Inferred from Mutant Phenotype

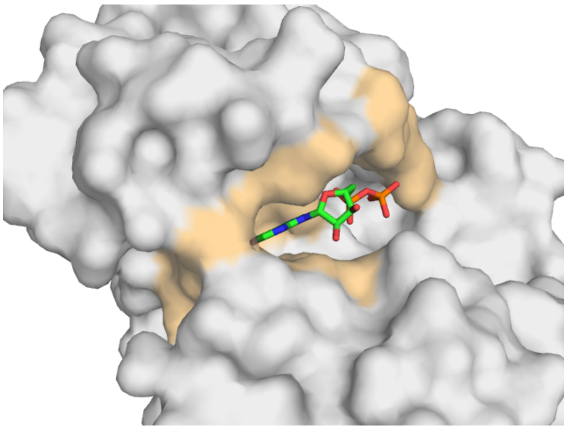

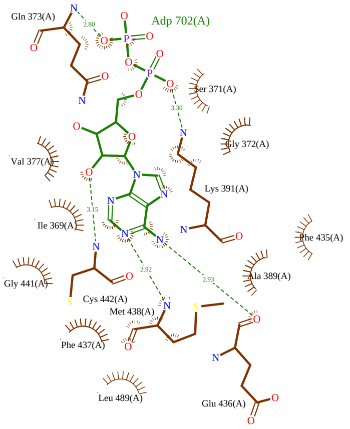

protein	amino acids contacts (binding site)
PDB:4m15:chainA:Q08881	I S G Q V A K F E F M G C L
sp:Q08881:ITK_HUMAN	I S G Q V A K F E F M G C L
tr:D4A7W7:D4A7W7_RAT	I S G Q V A K F E F M G C L
tr:Q8CAW3:Q8CAW3_MOUSE	I S G Q V A K F E F M G C L
tr:A1A560:A1A560_MOUSE	I S G Q V A K F E F M G C L
tr:Q5STT8:Q5STT8_MOUSE	I S G Q V A K F E F M G C L
sp:Q03526:ITK_MOUSE	I S G Q V A K F E F M G C L
sp:P08630:BTKL_DROME	L S G Q V A K T E Y M G S L

	whole protein		domain-based		contact-based	
protein	ident	simil	ident	simil	ident	simil
PDB:4m15:chainA:Q08881	1.0	1.0	1.0	1.0	1.0	1.0
sp:Q08881:ITK_HUMAN	1.0	1.0	1.0	1.0	1.0	1.0
tr:D4A7W7:D4A7W7_RAT	0.94	0.97	0.97	0.99	1.0	1.0
tr:Q8CAW3:Q8CAW3_MOUSE	0.53	0.55	0.97	0.99	1.0	1.0
tr:A1A560:A1A560_MOUSE	0.94	0.98	0.97	0.99	1.0	1.0
tr:Q5STT8:Q5STT8_MOUSE	0.94	0.98	0.97	0.99	1.0	1.0
sp:Q03526:ITK_MOUSE	0.93	0.97	0.97	0.99	1.0	1.0
sp:P08630:BTKL_DROME	0.35	0.62	0.55	0.85	0.71	0.84

Btk29A (FBgn0003502) associated phenotypes
cell shape defective, lethal - all die before end of first instar larval stage, mating defective, partially, short lived, size defective, some die during first instar larval stage

(Information from FlyBase)

Btk29A (UniProt:P08630) annotation
Function: Required for proper ring canal development. Also required for the development of male genitalia and for adult survival. (PubMed:10330180, PubMed:9655810).
Cofactor: Zn(2+)Note=Binds 1 zinc ion per subunit. ;
Subcellular location: Note=Ring canals.
Tissue specificity: Ring canals in the egg chambers and imaginal disks of third-instar larvae. (PubMed:10330180, PubMed:3110602, PubMed:9655810).
Developmental stage: Expressed both maternally and zygotically. Predominantly in early to middle embryogenesis, in larvae and adult females. (PubMed:10330180, PubMed:3110602).
Disruption phenotype: Flies exhibit shortened copulatory duration (due to incomplete fusion of the left and right halves of the apodeme that holds the penis during copulation) and reduced adult-stage life span. (PubMed:10330180).

(Information from UniProt)

Heat shock protein 90

Best gene implication in ageing for this target family came from gene Q18688 annotated in UniProt release 2014_02. Annotation GO 8340 (determination of adult lifespan) was Inferred from Genetic Interaction

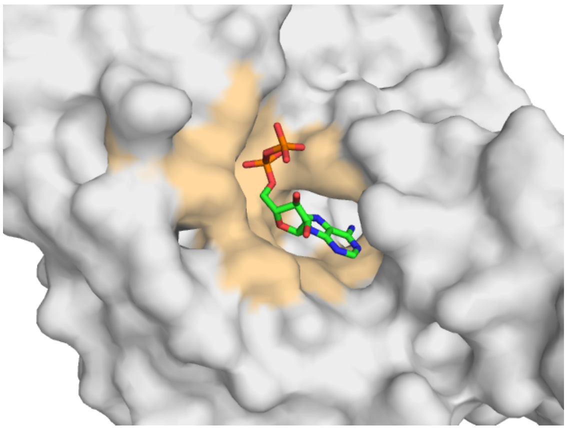

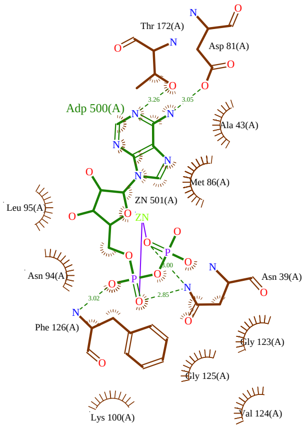

protein	amino acids contacts (binding site)											
PDB:4gqt:chainA:Q18688	N	A	D	M	N	L	K	G	V	G	F	T
tr:K9JA46:K9JA46_HUMAN	N	A	D	M	N	L	K	G	V	G	F	T
sp:P07900:HS90A_HUMAN	N	A	D	M	N	L	K	G	V	G	F	T
tr:Q6B437:Q6B437_RAT	N	A	D	M	N	L	K	G	V	G	F	T
sp:P82995:HS90A_RAT	N	A	D	M	N	L	K	G	V	G	F	T
tr:B7ZC50:B7ZC50_MOUSE	N	A	D	M	N	L	K	G	V	G	F	T
tr:Q8C2A9:Q8C2A9_MOUSE	N	A	D	M	N	L	K	G	V	G	F	T
tr:Q3TKB9:Q3TKB9_MOUSE	N	A	D	M	N	L	K	G	V	G	F	T
tr:Q3TKG0:Q3TKG0_MOUSE	N	A	D	M	N	L	K	G	V	G	F	T
sp:P07901:HS90A_MOUSE	N	A	D	M	N	L	K	G	V	G	F	T
tr:Q80Y52:Q80Y52_MOUSE	N	A	D	M	N	L	K	G	V	G	F	T
sp:P02828:HSP83_DROME	N	A	D	M	N	L	K	G	V	G	F	T
sp:Q18688:HSP90_CAEEL	N	A	D	M	N	L	K	G	V	G	F	T
sp:P02829:HSP82_YEAST	N	A	D	M	N	L	K	G	V	G	F	T

protein	whole protein ident	simil	domain-based ident	simil	contact-based ident	simil
PDB:4gqt:chainA:Q18688	1.0	1.0	1.0	1.0	1.0	1.0
tr:K9JA46:K9JA46_HUMAN	0.74	0.89	0.76	0.94	1.0	1.0
sp:P07900:HS90A_HUMAN	0.74	0.89	0.76	0.94	1.0	1.0
tr:Q6B437:Q6B437_RAT	0.18	0.22	0.62	0.78	1.0	1.0
sp:P82995:HS90A_RAT	0.73	0.89	0.76	0.94	1.0	1.0
tr:B7ZC50:B7ZC50_MOUSE	0.2	0.25	0.63	0.79	1.0	1.0
tr:Q8C2A9:Q8C2A9_MOUSE	0.24	0.31	0.76	0.94	1.0	1.0
tr:Q3TKB9:Q3TKB9_MOUSE	0.56	0.67	0.76	0.94	1.0	1.0
tr:Q3TKG0:Q3TKG0_MOUSE	0.56	0.67	0.76	0.94	1.0	1.0
sp:P07901:HS90A_MOUSE	0.73	0.89	0.76	0.94	1.0	1.0
tr:Q80Y52:Q80Y52_MOUSE	0.73	0.89	0.76	0.94	1.0	1.0
sp:P02828:HSP83_DROME	0.74	0.9	0.82	0.95	1.0	1.0
sp:Q18688:HSP90_CAEEL	1.0	1.0	1.0	1.0	1.0	1.0
sp:P02829:HSP82_YEAST	0.6	0.85	0.66	0.9	1.0	1.0

Hsp83 (FBgn0001233) associated phenotypes
cell cycle defective, cell polarity defective, developmental rate defective, female semi-fertile, lethal - all die before end of pupal stage, lethal - all die during third instar larval stage, partially lethal, partially lethal - majority die, short lived

(Information from FlyBase)

Hsp83 (UniProt:P02828) annotation
Function: Molecular chaperone that promotes the maturation, structural maintenance and proper regulation of specific target proteins involved for instance in cell cycle control and signal transduction. Undergoes a functional cycle that is linked to its ATPase activity. This cycle probably induces conformational changes in the client proteins, thereby causing their activation. Interacts dynamically with various co-chaperones that modulate its substrate recognition, ATPase cycle and chaperone function. Together with Hop and piwi, mediates canalization, also known as developmental robustness, likely via epigenetic silencing of existing genetic variants and suppression of transposon-induced new genetic variation. Required for piRNA biogenesis by facilitating loading of piRNAs into PIWI proteins. (PubMed:21186352, PubMed:22902557).
Subunit: Homodimer (By similarity). Forms a complex with Hop and piwi; probably Hop mediates the interaction between piwi and Hsp83. Interacts with shu. (, PubMed:21186352, PubMed:22902557).
Subcellular location: Cytoplasm.
Induction: In contrast to other major heat shock proteins, this one is also expressed at normal growth temperatures. It is also developmentally expressed during oogenesis.
Domain: The TPR repeat-binding motif mediates interaction with TPR repeat-containing proteins.

(Information from UniProt)

daf-21 (WBGene00000915) associated phenotypes
chemotaxis variant, dauer constitutive, dauer recovery variant, larval lethal, lethal, odorant chemosensory response variant, sluggish, sterile

(Information from WormBase)

daf-21 (UniProt:Q18688) annotation
Function: Molecular chaperone that promotes the maturation, structural maintenance and proper regulation of specific target proteins involved for instance in cell cycle control and signal transduction. Undergoes a functional cycle that is linked to its ATPase activity. This cycle probably induces conformational changes in the client proteins, thereby causing their activation. Interacts dynamically with various co-chaperones that modulate its substrate recognition, ATPase cycle and chaperone function. By stabilizing the receptor-type guanylate cyclase daf-11 or another signal transduction component that regulates cGMP levels, plays a role in dauer formation and chemotaxis to non-volatile and volatile attractants detected by AWC sensory neurons

(PubMed:10790386, PubMed:7828815). Participates in the control of cell cycle progression at the prophase/metaphase transition in oocyte development by ensuring the activity of wee-1.3 kinase, which negatively regulates cdk-1 through its phosphorylation (PubMed:16466390). Regulates yap-1 nuclear export after heat shock treatment (PubMed:23396260). (PubMed:10790386, PubMed:16466390, PubMed:23396260, PubMed:7828815).
Subunit: Homodimer. (UniProtKB:P08238).
Subcellular location: Cytoplasm, perinuclear region (PubMed:12950278). Note=Perinuclear region of somatic cells.
Tissue specificity: In the embryo comma stage, expression is strongly detected in cells of the head region and less so in other areas. In early larvae, expressed in postembryonic germ cells derived from Z2 and Z3 cells and the head region, in both hermaphrodites and males. Under heat stress conditions, larval expression is not only detected in germ cells, but also all over the body. In adult hermaphrodites, expression is localized uniquely in the germ cells. (PubMed:12950278).
Developmental stage: Highly expressed throughout development. (PubMed:10790386).
Domain: The TPR repeat-binding motif mediates interaction with TPR repeat-containing proteins.

(Information from UniProt)

Glycogen synthase kinase-3 beta

Best gene implication in ageing for this target family came from gene Q6FI27 via mapping the annotation from Ensembl ENSG00000082701 via mapping the annotation from EntrezGene 2932 via mapping the annotation from GenAgeHuman 0097 annotated in GenAge release 17.

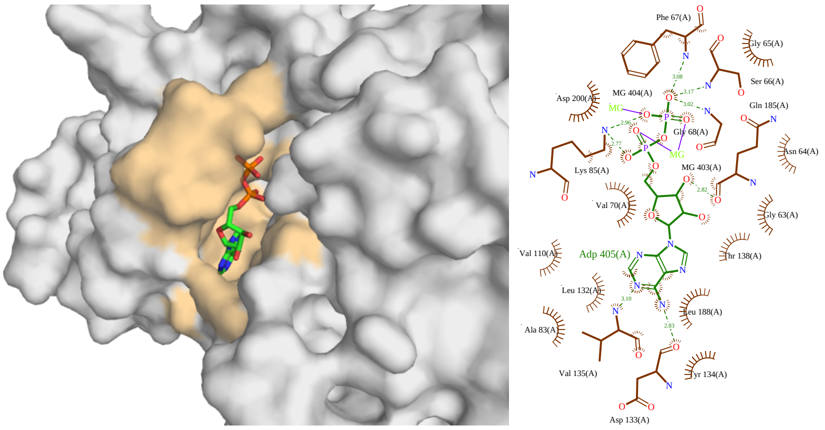

protein	amino acids contacts (binding site)
PDB:4nu1:chainA:Q9WV60	G N G S F G V A K V L D Y V T R Q N L D
tr:Q6FI27:Q6FI27_HUMAN	G N G S F G V A K V L D Y V T R Q N L D
sp:P49841:GSK3B_HUMAN	G N G S F G V A K V L D Y V T R Q N L D
sp:P18266:GSK3B_RAT	G N G S F G V A K V L D Y V T R Q N L D
tr:Q5KU03:Q5KU03_MOUSE	G N G S F G V A K V L D Y V T R Q N L D
sp:Q9WV60:GSK3B_MOUSE	G N G S F G V A K V L D Y V T R Q N L D
tr:E9QAQ5:E9QAQ5_MOUSE	G N G S F G V A K V L D Y V T R Q N L D
tr:B6IDN4:B6IDN4_DROME	G N G S F G V A K V L E Y I T K Q N L D
sp:P18431:SGG_DROME	G N G S F G V A K V L E Y I T K Q N L D
tr:A4V3W2:A4V3W2_DROME	G N G S F G V A K V L E Y I T K Q N L D
tr:A4V3W1:A4V3W1_DROME	G N G S F G V A K V L E Y I T K Q N L D
tr:Q0KHW6:Q0KHW6_DROME	G N G S F G V A K V L E Y I T K Q N L D
tr:E6PBX9:E6PBX9_DROME	G N G S F G V A K V L E Y I T K Q N L D
tr:H5V852:H5V852_DROME	G N G S F G V A K V L E Y I T K Q N L D
sp:Q9U2Q9:GSK3_CAEEL	G N G S F G V A K V L E Y V T R Q N L D
sp:P50873:MRK1_YEAST	G H G S F G V A K V L D Y M S Q Q N L D

	whole protein		domain-based		contact-based	
protein	ident	simil	ident	simil	ident	simil
PDB:4nu1:chainA:Q9WV60	1.0	1.0	1.0	1.0	1.0	1.0
tr:Q6FI27:Q6FI27_HUMAN	0.99	1.0	1.0	1.0	1.0	1.0
sp:P49841:GSK3B_HUMAN	0.99	1.0	1.0	1.0	1.0	1.0
sp:P18266:GSK3B_RAT	1.0	1.0	1.0	1.0	1.0	1.0
tr:Q5KU03:Q5KU03_MOUSE	1.0	1.0	1.0	1.0	1.0	1.0
sp:Q9WV60:GSK3B_MOUSE	1.0	1.0	1.0	1.0	1.0	1.0
tr:E9QAQ5:E9QAQ5_MOUSE	0.97	0.97	0.96	0.96	1.0	1.0
tr:B6IDN4:B6IDN4_DROME	0.58	0.71	0.85	0.96	0.85	0.95
sp:P18431:SGG_DROME	0.61	0.74	0.85	0.96	0.85	0.95
tr:A4V3W2:A4V3W2_DROME	0.61	0.74	0.85	0.96	0.85	0.95
tr:A4V3W1:A4V3W1_DROME	0.54	0.66	0.85	0.96	0.85	0.95
tr:Q0KHW6:Q0KHW6_DROME	0.5	0.63	0.85	0.96	0.85	0.95
tr:E6PBX9:E6PBX9_DROME	0.19	0.26	0.54	0.7	0.85	0.95
tr:H5V852:H5V852_DROME	0.28	0.35	0.85	0.96	0.85	0.95
sp:Q9U2Q9:GSK3_CAEEL	0.59	0.72	0.81	0.95	0.95	0.98
sp:P50873:MRK1_YEAST	0.32	0.58	0.53	0.84	0.8	0.91

sgg (FBgn0003371) associated phenotypes

cell non-autonomous, cell polarity defective, chemical sensitive, circadian rhythm defective, developmental rate defective, germline clone, heat sensitive, lethal - all die before end of P-stage, lethal - all die before end of pupal stage, maternal effect, meiotic cell cycle defective, mitotic cell cycle defective, neuroanatomy defective, neurophysiology defective, non-rescuable maternal effect, planar polarity defective, rescuable maternal effect, short lived, smell perception defective, somatic clone, some die during pharate adult stage, some die during pupal stage, some die during third instar larval stage

(Information from FlyBase)

sgg (UniProt:P18431) annotation

Function: Required for several developmental events such as syncytial blastoderm formation and embryonic segmentation. Is involved in transcriptional regulation. Required for arm phosphorylation. Wg signaling operates by inactivating the sgg repression of en autoactivation. Negatively controls the neuromuscular junction (NMJ) growth in presynaptic motoneurons. Plays a role in the regulation of microtubule dynamics and actin cytoskeleton during embryogenesis. Required for phosphorylation of sra in activated eggs. Essential for completion of meiosis, possibly by triggering calcineurin activation via sra phosphorylation. Phosphorylates microtubule-associated protein futsch in axons. (PubMed:15269269, PubMed:16570248, PubMed:16949836, PubMed:2113617, PubMed:2118107, PubMed:22421435, PubMed:7529201, PubMed:8467811).
Subunit: Interacts with cos. (PubMed:1335365,PubMed:15691767).

Subcellular location: Cytoplasm. Nucleus. Cytoplasm, cytoskeleton, microtubule organizing center, centrosome. Cytoplasm, cell cortex. Cell junction, synapse. Cell projection, axon. Note=In syncytial embryos, detected at the centrosomes throughout the cell cycle, and in the mitotic spindle and pseudocleavage furrows invaginating from the cell cortex during mitosis. Concentrated at the growing end of membranes during the cellularization process. After cellularization, localized to the centrosomes during mitosis and to the nucleus at the end of telophase. Enriched in the presynaptic side of the neuromuscular junction, with some signal detected also in axonal branches.
Tissue specificity: Expressed in ovaries and activated eggs (at protein level). Expression is over all the embryo at all stages, no local accumulation is observed. (PubMed:2118107, PubMed:22421435, PubMed:8467811).
Developmental stage: Isoform SGG46 is expressed at low levels in 12-24 hours embryos. Isoform Zygotic and isoform SGG39 are expressed in 12-24 hours embryos and present throughout the larval, pupal and adult stages (at protein level). Isoform Zygotic is expressed maternally and zygotically but reduced throughout later embryonic development. Expression persists throughout larval stages. (PubMed:2118107, PubMed:8467811).
Disruption phenotype: Mutants display an overdeveloped neuromuscular junction (NMJ), with the number of boutons greatly increased. (PubMed:15269269).

(Information from UniProt)

gsk-3 (WBGene00001746) associated phenotypes

axon morphology variant, larval lethal, lethal, locomotion variant, sterile

(Information from WormBase)

gsk-3 (UniProt:Q9U2Q9) annotation

Function: Phosphorylates oma-1, a regulator of the oocyte-to- embryo transition, enabling its degradation. Phosphorylates skn-1, preventing it from accumulating in nuclei and thus inhibiting phase II gene expression in the oxidative stress defense. Involved in mesendoderm specification and mitotic spindle orientation in EMS blastomeres. Thought to be a branch point in these processes as proteins downstream are not required. Negatively regulates Wnt signaling in vulval precursor cells and acts as a Wnt-independent repressor of med-1 and med-2 in the C lineage inhibiting mesoderm development. Required for normal lifespan and LiCl-induced lifespan extension. (PubMed:10444600, PubMed:11463373, PubMed:15572126, PubMed:16251270, PubMed:16289132, PubMed:16343905, PubMed:16930586, PubMed:17959600).
Subunit: Monomer (By similarity). Interacts with axl-1. (UniProtKB:Q9WV60, PubMed:17601533).
Disruption phenotype: Worms exhibit defects in endoderm specification and mitotic spindle alignment. Mutants show reduced degradation of oma-1 and have shortened lifespan. (PubMed:10444600).

(Information from UniProt)
